# Ultrathin structures derived from interfacially modified polymeric nanocomposites to curb electromagnetic pollution†

**DOI:** 10.1039/d0na01071e

**Published:** 2021-03-08

**Authors:** Kumari Sushmita, Petr Formanek, Dieter Fischer, Petra Pötschke, Giridhar Madras, Suryasarathi Bose

**Affiliations:** Centre for Nanoscience and Engineering, Indian Institute of Science Bangalore-560012 India; Leibniz-Institut für Polymerforschung Dresden e. V. (IPF) Dresden-01069 Germany; Interdisciplinary Centre for Energy Research, Indian Institute of Science Bangalore-560012 India; Department of Materials Engineering, Indian Institute of Science Bangalore-560012 India sbose@iisc.ac.in

## Abstract

The use of electronic devices and wireless networks is increasing rapidly, and electromagnetic (EM) pollution remediation remains a challenge. We employed a unique approach to fabricate two ultrathin (approx. 53 μm) multilayered assemblies to address this. By sequentially stacking thin films of polyvinylidene difluoride (PVDF) and polycarbonate (PC) nanocomposites and interfacially locking them with a mutually miscible polymer (PMMA, polymethyl methacrylate), materials with enhanced structural properties and electromagnetic interference (EMI) shielding performance can be designed. Utilizing reduced graphene oxide (rGO) and molybdenum disulfide (MoS_2_) as a template, ferrite was grown on the surface to design two different nanohybrid structures (rGO–Fe_3_O_4_ and MoS_2_–Fe_3_O_4_). PVDF was composited with either rGO–Fe_3_O_4_ or MoS_2_–Fe_3_O_4_, and multiwall carbon nanotubes (CNTs) were dispersed in the PC component. As PC and PVDF are immiscible, their poor interface would result in inferior structural properties, which can be challenging in designing EMI shielding materials due to cyclic thermal fatigue. Hence, PMMA is sandwiched to interfacially stitch the components (PC and PVDF) and improve interfacial adhesion. This was confirmed using SEM/EDS and Raman mapping/imaging. The mechanical stability of the multilayered assemblies was characterized using a dynamic mechanical analyzer (DMA), and the storage modulus was found to be as high as 2767 MPa at 40 °C (@constant frequency and strain amplitude), for the multilayered film with rGO–Fe_3_O_4_ in PVDF, PMMA as a sandwich layer and CNTs in PC. A typical assembly of 9 multilayers (∼480 μm) with rGO–Fe_3_O_4_ in PVDF, and CNTs in PC, and interfacially stitched with PMMA gave rise to a high EMI shield effectiveness (SE_T_) of −26.3 dB @ 26.5 GHz. This unique arrangement of a multilayered assembly suppressed EMI primarily by absorption.

## Abstract 2

Electronic devices and modern communication networks have enhanced electromagnetic (EM) pollution, necessitating finding robust alternate materials to suppress it.

## Graphical abstract

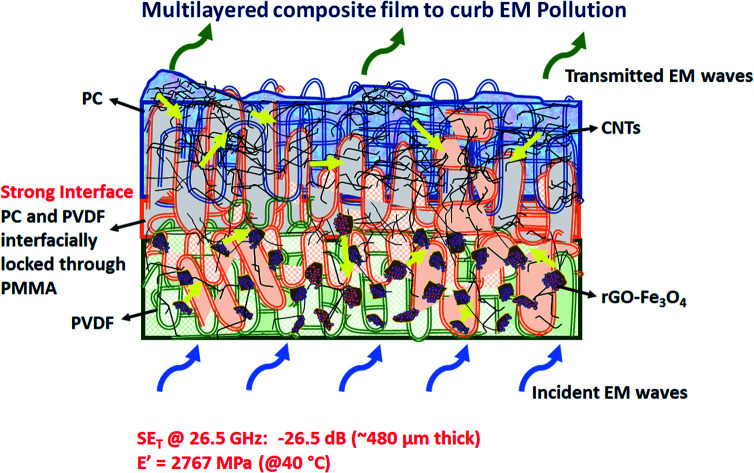

## 1. Introduction

With the increasing development of high-speed electronic devices, electromagnetic interference (EMI) has become a significant concern. To protect sensitive electronic circuits from unwanted electromagnetic (EM) radiation, EMI shielding materials need to be developed. EMI shielding refers to minimizing the transmission of incoming EM radiations by introducing a shielding material that can obstruct the waves by reflection and/or adsorption. Research in the last couple of decades has focused on metals and polymer nanocomposites as a solution to EM pollution.^1–3^ Polymer nanocomposites have an added advantage over metals due to their ease of processing, corrosion resistivity, and low density.^3,4^ Both intrinsically conducting and insulating polymers have been extensively explored for EMI shielding applications.^2,3,5–8^ The polymer acts as a matrix for incorporating nanoparticles that interact with the EM waves and attenuate them *via* reflection, absorption, or multiple reflections. The most common and useful fillers for EMI shielding applications include carbon-based nanofillers such as carbon nanotubes (CNTs), reduced graphene oxide (rGO), graphene platelets, carbon black, *etc.*^5–7^ Magnetically lossy or dielectrically lossy fillers also interact and suppress the incoming EM radiation *via* absorption.^9^

Herein, two functional hybrid structures were synthesized using reduced graphene oxide (rGO) and molybdenum disulfide (MoS_2_) as templates; rGO–Fe_3_O_4_ and MoS_2_–Fe_3_O_4_. Several studies have been performed on rGO, MoS_2_, and Fe_3_O_4_ based materials for EMI shielding applications.^10,11^ For a polymer to act as an EMI shield, conducting and magnetically/dielectrically lossy nanoparticles have to be incorporated. In our work, we have used multiwalled carbon nanotubes (CNTs) as a conducting filler,^12^ given their ability to form a percolated network at a relatively lower concentration on account of their high aspect ratio, and incorporated rGO–Fe_3_O_4_ and MoS_2_–Fe_3_O_4_ as the lossy counterpart. Fe_3_O_4_ is a ferrimagnetic semiconducting material.^13^ In the case of rGO, the extent of reduction of GO decides the properties of rGO in terms of conductivity as well as defects, which play a significant role in EMI shielding. MoS_2_ is semiconducting and known to be dielectrically lossy.^14–17^

The dispersion of nanoparticles in a polymer matrix and the interaction between the polymer and nanoparticles play a crucial role in EMI shielding performance. In addition to this, several studies have focused on optimizing nanoparticle design for better EMI shielding properties. This includes synthesizing heterostructured nanoparticles such as core–shell structures and studying their effect on EMI shielding performance by varying the interfaces/interfacial properties.^18–20^ Another important aspect is the polymer blends/nanocomposite architecture, which includes selective localization of nanoparticles, the multilayer architecture with nanofillers, *etc.*^4,21–23^

Multilayered architecture is an exciting research domain known to enhance the shielding effectiveness (SE_T_) at lower thicknesses primarily due to multiple scattering and interfacial polarization, thus giving an advantage over conventional shields. Zhang *et al.*^23^ reported a multilayered assembly of regenerated cellulose as the supporting substrate and polyethylene oxide (PEO)/CNT as the EMI shielding layer. PEO enhanced the interfacial adhesion between the CNTs and the cellulose layers due to its favorable compatibility with cellulose chains. Compression molding was used to sandwich the PEO/CNT (1 : 4) layer between the cellulose layer, and EMI shielding measurements were performed in the X-band. For a film thickness of 150 μm, the layered structure showed a SE_T_ value of −35 dB compared to −20 dB demonstrated by the plain structured composite, which was prepared by direct mixing of CNTs into cellulose solutions. Xu *et al.*^22^ reported a highly conductive sandwich structure of a nylon/nickel film prepared by the electroless deposition method. For a film thickness of 100 μm, SE_T_ was reported to be −77 dB in the X-band. Wang *et al.*^24^ fabricated nitrogen-doped graphene multilayer films by thermal annealing of stacked graphene oxide/copper phthalocyanine (GO/CuPc) multilayer films. The maximum SE_T_ value of −55.2 dB was obtained for a shield thickness of 0.47 mm in the X-band. Biswas *et al.*^21^ stacked individual functional nanocomposites consisting of PC/PVDF blends with CNTs, functionalized CNTs, and CNTs conjugated with flower-like Fe_3_O_4_ nanoclusters for effective EMI shielding performance. This compression-molded stack showed a shielding efficiency of −64 dB at 18 GHz frequency for a shield thickness of 0.9 mm. In another work by Biswas *et al.*,^4^ a multilayered assembly with PC/PVDF + 3 wt% CNT sandwiched between PVDF + 3 wt% CNT–MnO_2_ + rGO/Fe was constructed. For a shield thickness of 0.9 mm, the EMI shielding effectiveness was approximately −57 dB in the frequency range of 8–18 GHz.

The literature also suggests that it is not only the type of nanofiller but also the type of polymer and sequential stacking of these layers that become important in designing a robust multilayered assembly for EMI shielding applications. The choice of polymers in our work is based on the existing PVDF/PMMA and PMMA/PC blend systems in the literature. It is reported that PMMA decreases the interfacial tension and enhances the interfacial adhesion between PC and PVDF.^25–28^ It is miscible with both PC and PVDF and is known to act as a common solvent in the melt state.^29–33^ Also, few articles report that PVDF/PMMA is miscible in the solution state (forms homogeneous solution under certain conditions) due to the interaction between the H-atom of PVDF and the O-atom in the carbonyl group of PMMA, but they phase separate after solvent evaporation.^30,34^ Besides, Sharma *et al.*^35^ reported that the strong intermolecular interaction in the melt state of the PVDF/PMMA blend system not only arises from hydrogen bonding between carbonyl groups in PMMA and the –CH_2_ groups of PVDF but also the dipole–dipole interactions between –CH_2_ of PMMA and CF_2_ in PVDF. Similarly, there are reports where PMMA/PC results in a homogeneous solution under certain conditions.^36,37^ Blend miscibility (above 200 °C) between PMMA and PC can be explained using various chemical reactions such as unzipping of PMMA, the attack of PMMA macroradicals on PC chains, *trans*-esterification of the PMMA ester pendant group with PC, and termoxidative branching leading to graft copolymer inducing re-homogenization at high temperature.^29^ Few other articles on PMMA/PC blend systems confirm that the specific interactions between the ester group of PMMA and the phenyl ring of PC under certain conditions play a significant role in miscibility.^37,38^

This work's uniqueness lies in polymer selection and the processing technique used for the construction of the multilayered EMI shielding stack. Herein a doctor blade setup has been used to stack the PVDF, PMMA, and PC layers sequentially. Thus, PMMA was chosen to stitch PC and PVDF layers interfacially. We carried out a templated growth of Fe_3_O_4_ on rGO and MoS_2_ for uniform dispersion. By rationally stitching materials with different characteristics as ‘interfacially locking, PMMA’ between a conducting layer (PC with CNTs) and lossy layer (PVDF with either rGO–Fe_3_O_4_ or MoS_2_–Fe_3_O_4_), the incoming EM radiations can be blocked.

## 2. Experimental section

### 2.1 Materials

Polycarbonate (PC) (Lexan 143 R, MFI-11 g/10 min, *M*_w_ = 37 492, *M*_n_ = 20 642, PDI = 1.8) was procured from Sabic. Polymethylmethacrylate (PMMA) with a molecular weight (*M*_w_) of 123 000 g mol^−1^, Mn of 56 981 g mol^−1^, and a polydispersity of 2.1 was bought from Gujpol. Polyvinylidene fluoride (PVDF) was purchased from Arkema (Kynar 761 grade) with a *M*_w_ of 440 000 g mol^−1^. A pristine multiwalled carbon nanotube (CNT) material, NC7000 (length 1.5 μm and diameter 9.5 nm), was supplied by Nanocyl SA (Belgium). Graphene oxide (GO) powder (BTGOX) was procured from BT Corp. Ferric chloride hexahydrate (FeCl_3_·6H_2_O) LR 98% was obtained from Thomas Baker. Hydrazine hydrate (H_4_N_2_·H2O) (99%), ethylene glycol (C_2_H_6_O_2_), and urea (NH_2_CONH_2_) were procured from SDFCL. Sodium molybdate dihydrate (Na_2_MoO_4_·2H_2_O) and thiourea (NH_2_CSNH_2_) were procured from MERCK. Chloroform and dimethylformamide (DMF) were procured from SDFCL. Analytical grade absolute ethanol was procured from Changshu Hongsheng Fine Chemical Co., Ltd.

### 2.2 Synthesis of nanoparticles

#### 2.2.1 Synthesis of rGO–Fe_3_O_4_

**Scheme 1:**
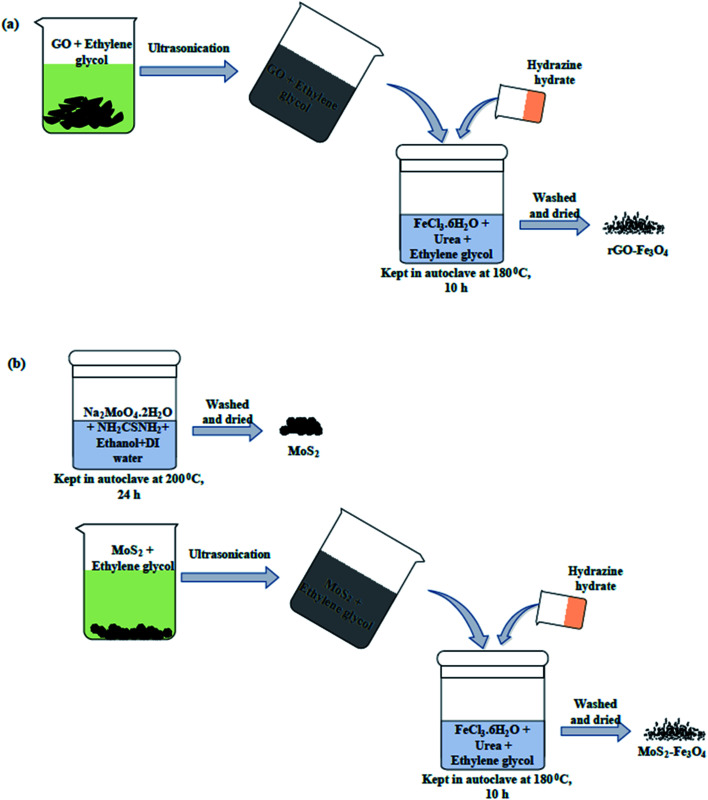
Synthesis protocol of (a) rGO–Fe_3_O_4_ and (b) MoS_2_–Fe_3_O_4_.

A solvothermal approach was used for the synthesis of rGO–Fe_3_O_4_ nanoparticles^39^ (Scheme 1a). As per the typical synthesis procedure, 200 mg of GO was dispersed in 60 mL of ethylene glycol and probe sonicated for 20 min to eliminate the primary agglomeration. Further, the reaction mixture was bath-sonicated for 45 min to eliminate the secondary agglomeration. The GO dispersion was then added to the solution of FeCl_3_·6H_2_O, prepared by adding 500 mg of FeCl_3_·6H_2_O and 1 g of urea in 20 mL of ethylene glycol. The reaction mixture was stirred well and transferred into a 100 mL Teflon-lined stainless-steel autoclave. Hydrazine hydrate (3 mL) was immediately added, and the autoclave was sealed manually. The reaction was performed in a preheated oven at 180 °C for 10 h. The autoclave was then allowed to cool down to room temperature, and the black powder of rGO–Fe_3_O_4_ was separated using centrifugation. Lastly, it underwent several washing cycles with DI water and ethanol before drying and storing for further usage.

#### 2.2.2 Synthesis of MoS_2_–Fe_3_O_4_

A two-step solvothermal method was used to synthesize MoS_2_–Fe_3_O_4_ nanoparticles (Scheme 1b). Firstly, MoS_2_ nanoparticles were synthesized by dissolving 2.5 g of Na_2_MoO_4_·2H_2_O and 1.55 g of NH_2_CSNH_2_ (molar ratio of 1 : 2) in an ethanol: DI water mixture (volume ratio of 1 : 1). The reaction mixture was prepared in a 50 mL autoclave, and the reaction was carried out for 24 h at 200 °C. The obtained powder was washed with DI water and ethanol before it was dried and stored. Using a similar procedure to previously described (used for the synthesis of rGO–Fe_3_O_4_), the synthesis of MoS_2_–Fe_3_O_4_ was carried out by dispersing MoS_2_ in place of rGO. Unlike rGO, the probe sonication of MoS_2_ flowers wasn't performed to avoid the breakage of MoS_2_ flowers. But bath sonication of MoS_2_–ethylene glycol was carried out for good dispersion. The rest of the procedure was the same.

### 2.3 Preparation of the multilayered structure of PVDF/PMMA/PC with nanofillers

#### 2.3.1 The conceptual basis for the choice of polymers and solvents

**Scheme 2:**
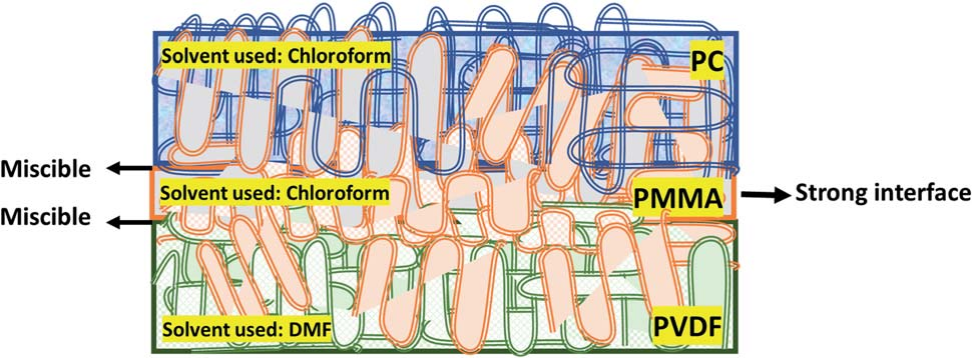
Schematic showing the conceptual basis for the fabrication of the multilayered assembly of PVDF/PMMA/PC based on the miscibility of individual polymers.

As PMMA decreases the interfacial tension and enhances the PC/PVDF system's interfacial adhesion, a multilayered assembly of PVDF/PMMA/PC was chosen. This technique's concept is well explained in Scheme 2 and further supported by the digital images in Fig. S1.† The polymer–solvent pair was decided after a couple of optimization experiments based on the theoretical calculations in Table S1† and the visual inspection of the obtained film. In addition, the essential characterization of the commercial polymers (PVDF, PMMA and PC) is shown in Fig. S2.†

#### 2.3.2 Experimental procedure for the fabrication of the multilayered assembly with fillers

PVDF-hybrid nanoparticle (rGO–Fe_3_O_4_ or MoS_2_–Fe_3_O_4_) dispersions (10 wt% nanoparticles), PMMA solution, and PC–CNT dispersion (3 wt% CNT) were prepared by dissolving each of them in dimethylformamide (DMF), chloroform, and chloroform, respectively.

Preparation of PVDF-hybrid nanoparticle (rGO–Fe_3_O_4_ or MoS_2_–Fe_3_O_4_) solution: 1.8 g PVDF was dissolved in 7 mL DMF, and 200 mg of nanoparticles were dispersed in 3 mL DMF *via* bath sonication for 30 min. Shear mixing and magnetic stirring were avoided as Fe_3_O_4_ is magnetic. PVDF solution was then added dropwise to the nanoparticle dispersion, and the final dispersion was bath sonicated again for 20 min to obtain as homogeneous dispersion as possible.

Preparation of PMMA solution: PMMA solution was prepared by dissolving 3 g PMMA in 10 mL solvent.

Preparation of PC–CNT solution: 2.425 g PC was dissolved in 7 mL solvent. 75 mg CNTs were then added into 3 mL chloroform in a separate vial and bath sonicated. Since the viscosity was too high for casting, 10 mL chloroform was further added to the CNT dispersion, and bath sonication was carried out for 30 min. The PC solution was then added dropwise to the CNT dispersion and shear mixed at 9000 rpm for 45 min to obtain a homogeneous PC–CNT dispersion.

After preparing the three solutions/dispersions described in Section 2.3, the PVDF-hybrid nanoparticle dispersion was cast using a doctor blade setup (100 μm slit) and dried partially for 45 min at room temperature. Subsequently, the PMMA solution was cast using the doctor blade (200 μm slit) above the previous film and dried for 15 min at room temperature. Next, PC–CNT dispersion was cast using a doctor blade (300 μm slit) and dried for 15 min. The multilayered film was immersed in cold water for a few minutes and peeled off. It was then placed between two steel plates and dried in a vacuum oven at 60 °C. It is to be noted that this technique of sequential casting using increasing slit height (100 μm, 200 μm, 300 μm) was performed to obtain a 100 μm thick layer, each of PVDF-hybrid nanoparticles, PMMA and PC–CNT. However, after solvent evaporation, the actual thickness obtained is less than the total expected thickness of 300 μm, as is later seen in the scanning electron micrographs.

**Scheme 3:**
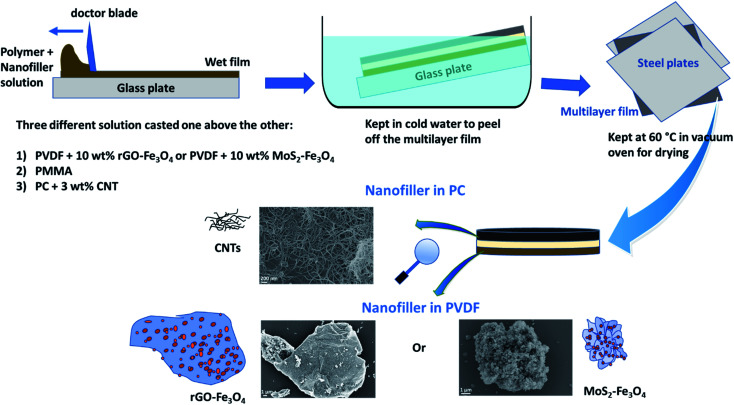
Schematic showing the preparation of the multilayered film.

The final multilayered stack is shown in Scheme 3. PVDF with hybrid nanoparticles (rGO–Fe_3_O_4_ or MoS_2_–Fe_3_O_4_) and PC–CNT composites are sandwiched using PMMA as an interfacial layer, and this multilayer is denoted as PVDF(nanofiller name)/M/PC. The multilayered assembly with rGO–Fe_3_O_4_ is represented as PVDF(rGO–Fe_3_O_4_)/M/PC, and MoS_2_–Fe_3_O_4_ is represented as PVDF(MoS_2_–Fe_3_O_4_)/M/PC hereon. It is to be noted that the multilayered assembly always has CNTs as a nanofiller in the PC layer, but for simplicity of nomenclature, we haven't explicitly mentioned it.

Control samples of PC–CNT were prepared by casting the dispersion of PC + 3 wt% CNT using a 300 μm slit, and the subsequent procedure was the same as shown in Scheme 3. The control samples of PVDF + rGO–Fe_3_O_4_ (10 wt% nanoparticles) and PVDF + MoS_2_–Fe_3_O_4_ (10 wt% nanoparticles) were also prepared by casting the respective dispersions individually using a 300 μm slit, but the film obtained was very brittle due to the high loading of nanoparticles, as shown in Fig. S5.† The control samples of single-layered PC + 3 wt% CNT, PVDF + 10 wt% rGO–Fe_3_O_4,_ and PVDF + 10 wt% MoS_2_–Fe_3_O_4_ using the doctor blade approach will be henceforth represented as PC(CNT), PVDF(rGO–rGO–Fe_3_O_4_), and PVDF(MoS_2_–Fe_3_O_4_) respectively.

## 3. Characterization

X-ray diffraction (XRD) analysis of the synthesized powder was carried out using an XPERT Pro from PANalytical. A Cu-Kα radiation source (*λ* = 1.5406 Å, 40 kV, and 30 mA) was used for analysis.

Transmission electron microscopy (TEM) images and the selected area diffraction (SAED) pattern of various nanoparticles were obtained using a Libra 120 TEM instrument. The morphology was analyzed by Raman microscopy using a RAMAN Imaging System WITEC alpha300R (using a 532 nm laser with a power of 5 mW and a 20× objective with a measuring point distance of 500 nm) and scanning electron microscopy (SEM) and energy-dispersive X-ray spectroscopy analysis (EDS) using a Carl Zeiss Ultra 55 or Carl Zeiss Ultra FE-SEM. Raman microscopy and SEM analysis of the cross-section of the multilayered assembly were performed after embedding it in epoxy and sectioning it using a microtome with a steel knife (for Raman microscopy, 20 μm thin sections) and ultramicrotome with a diamond knife (for SEM, block-face cuts). The SEM samples were coated with ∼10 nm of C to prevent charging in the electron beam. However, the SEM and EDS of nanoparticles were carried out with a few nm of gold coating over it. The differential scanning calorimetry (DSC) of polymers was performed in a copper pan using TA analysis (DSC Q2000) under a N_2_ atmosphere. Fourier transform infrared (FTIR) spectroscopy was performed using an FTIR instrument bought from PerkinElmer. % transmittance was obtained in the wavenumber range of 4000–650 cm^−1^ using universal attenuated total reflectance mode (ATR).

Magnetic measurements of the nanoparticles were performed using a physical property measurement system (9T) from Quantum Design. Room temperature AC conductivity studies were performed using an Alpha-A Analyser (Novocontrol, Germany) in a broad frequency range from 10^−1^ to 10^7^ Hz. Compression-molded discs (10 mm diameter and 1 mm thickness) were used as specimens for electrical conductivity measurements. Broadband (8.2–26.5 GHz) microwave shielding studies were performed using a keysight fieldfox microwave analyzer N9918A. *S*-parameters (*S*_11_, *S*_12_, *S*_21_, and *S*_22_) obtained from a vector network analyzer (VNA) were used to determine the total shielding effectiveness and shielding effectiveness due to reflection and absorption. Melt rheological analysis was performed using a Rheometer (model ARES G2) from TA Instruments, USA. The measurements were carried out under a constant strain of 1% in a nitrogen atmosphere. Two sweeps, between 0.1 and 100 rad s^−1^, forward and backward, were performed with the same filling, whereby the second one was used for interpretation. The following strain sweep was used to confirm that the selected strain was within the linear-viscoelastic range. Dynamic mechanical analysis (DMA) of the samples was performed using a DMA Q800. DMA samples were prepared by cutting a piece of size 2.2 cm × 0.63 cm from the multilayered thin film and were analyzed in tension mode as a function of temperature at a constant frequency of 1 Hz, constant oscillation amplitude of 3 μm, and strain% of 0.02, which lies in the viscoelastic region.

## 4. Results and discussion

### 4.1 Characterization of hybrid nanostructures

**Fig. 1:**
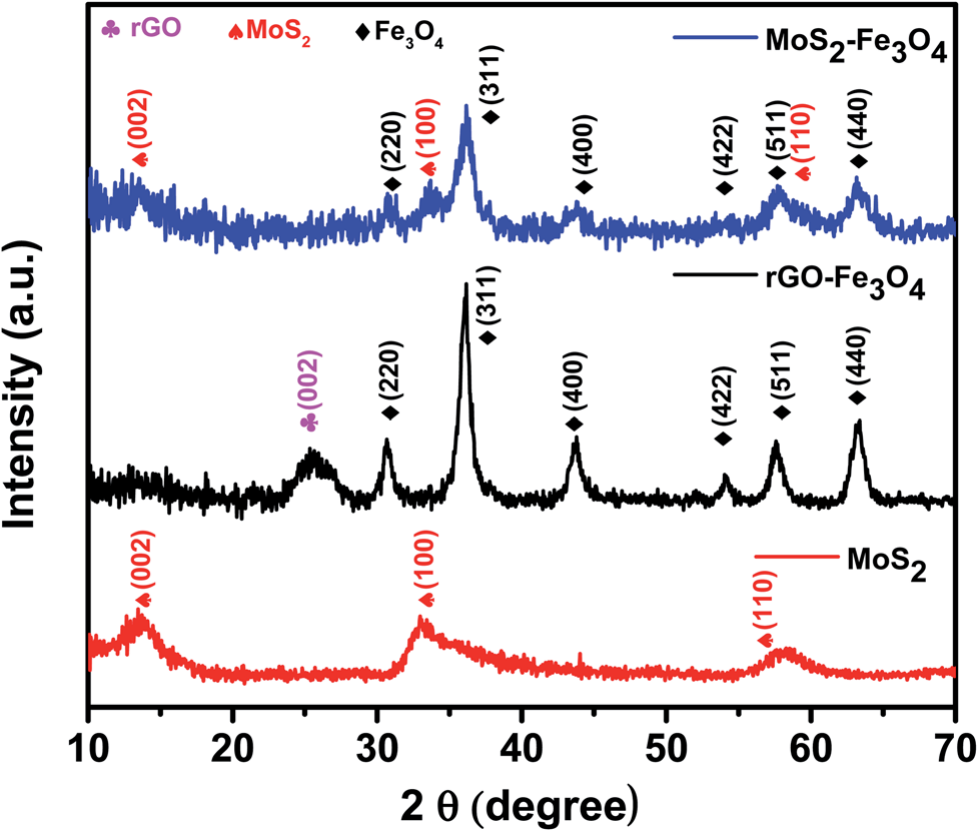
XRD pattern of MoS_2_, rGO–Fe_3_O_4,_ and MoS_2_–Fe_3_O_4_.

The X-ray powder diffraction (XRD) patterns of as-prepared MoS_2_, rGO–Fe_3_O_4,_ and MoS_2_–Fe_3_O_4_ are shown in Fig. 1. The XRD pattern of MoS_2_ shows its characteristic peak at 2*θ* values of 13.9°, 33°, and 58.3°, which correspond to the (002), (100), and (110) diffraction planes.^16,40^ These diffraction planes can be indexed to the hexagonal phases of MoS_2_. The XRD pattern of MoS_2_–Fe_3_O_4_ shows its characteristic peak at 2*θ* values of 13.6°, 30.7°, 33.3°, 36.2°, 43.8°, 54.2°, 57.8°, and 63.1°. Here the peaks at 30.7°, 36.2°, 43.8°, 54.2°, 57.8°, and 63.1° correspond to the cubic inverse spinel structure of Fe_3_O_4_, and the rest of the peaks correspond to the hexagonal phase of MoS_2_. The XRD pattern of rGO–Fe_3_O_4_ shows the characteristic peak of the (002) plane of rGO at 2*θ* ∼ 25.3°. The peaks at 2*θ* values of 30.7°, 36.2°, 43.8°, 54°, 57.6°, and 63.3° correspond to the diffraction planes of (220), (311), (400), (422), (511), and (440) and can be indexed to the cubic inverse spinel structure of Fe_3_O_4_.^39^

**Fig. 2:**
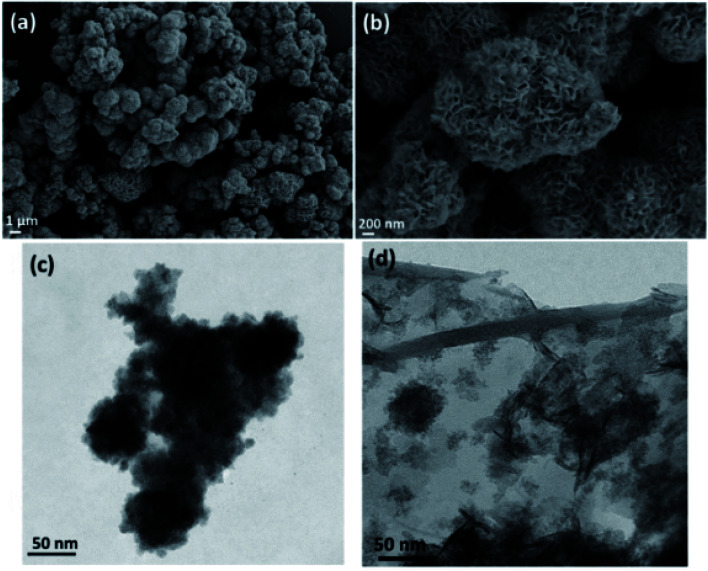
(a and b) SEM micrograph of MoS_2_, (c) TEM micrographs of MoS_2_–Fe_3_O_4_, and (d) TEM micrograph of rGO–Fe_3_O_4_.

Fig. 2 shows the SEM micrograph of MoS_2_ (Fig. 2a and b), and TEM micrograph of MoS_2_–Fe_3_O_4_ (Fig. 2c), and rGO–Fe_3_O_4_ (Fig. 2d), respectively. Fig. S3a and b† show the SEM micrograph of MoS_2_–Fe_3_O_4_, and Fig. S3c and d† show the SEM micrograph of rGO–Fe_3_O_4_. Fig. 2a and b illustrate a flower-like MoS_2_ structure of 0.3–5 μm dimensions. The MoS_2_ nanosheets self-assemble to form a thermodynamically and kinetically stable flower-like MoS_2_ structure during 24 h of a hydrothermal reaction. Fig. 2c, S3a, and b† show the morphology of MoS_2_–Fe_3_O_4_. We observe that the Fe_3_O_4_ nanoparticles have nucleated and grown on the MoS_2_ surface covering the entire petal-like structure. The size variation of the agglomerated chunks corresponds to 0.1–8 μm. The smaller sizes are more prominent in the TEM micrographs (Fig. S4a†). Fig. 2d, S3c, and d† show the rGO–Fe_3_O_4_ structure where spherical Fe_3_O_4_ nanoparticles decorate the rGO sheets well. Since both probe and bath sonication is used while synthesizing rGO–Fe_3_O_4_, the sheet obtained is of varying sizes from 0.7–20 μm, as evident from the low magnification TEM micrographs (Fig. S4b†). Additionally, Fig. S4c and d† show the SAED pattern of MoS_2_–Fe_3_O_4_ and rGO–Fe_3_O_4_ nanoparticles.

**Fig. 3:**
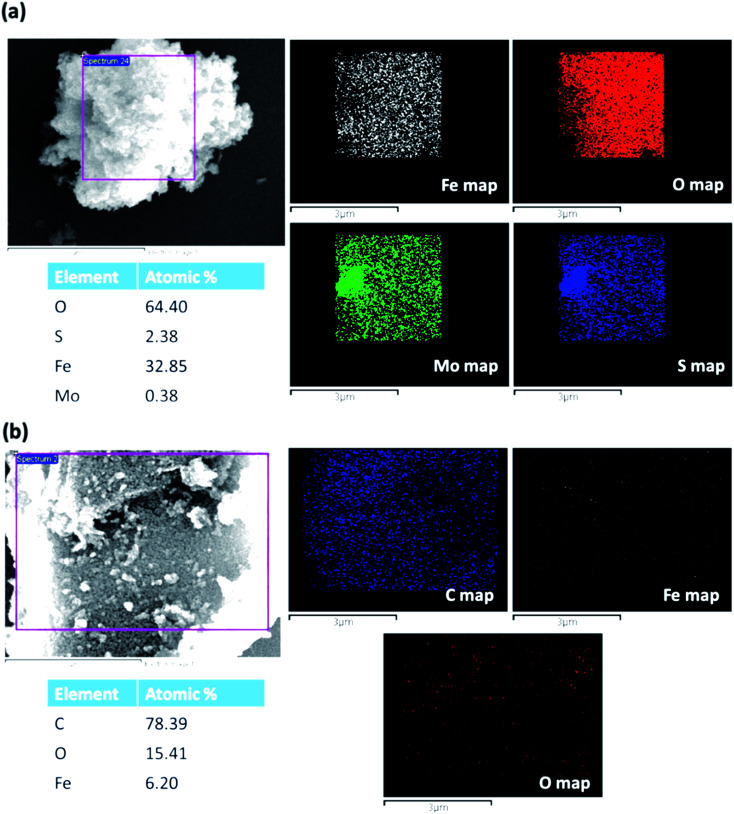
EDS map of (a) MoS_2_–Fe_3_O_4_ and (b) rGO–Fe_3_O_4_.

Fig. 3a shows the EDS map of MoS_2_–Fe_3_O_4_, and Fig. 3b shows the EDS mapping of rGO–Fe_3_O_4_. The map of MoS_2_–Fe_3_O_4_ and rGO–Fe_3_O_4_ shows the presence of individual elements. Though the weight ratios of GO/FeCl_3_·6H_2_O and MoS_2_/FeCl_3_·6H_2_O were both kept as 0.4 at the time of synthesis, we observe that the Fe atomic wt% is 32.8% in MoS_2_–Fe_3_O_4_ as compared to 6.2% in rGO–Fe_3_O_4_. However, it is to be noted that EDS is more of a qualitative technique than a quantitative one.

**Fig. 4:**
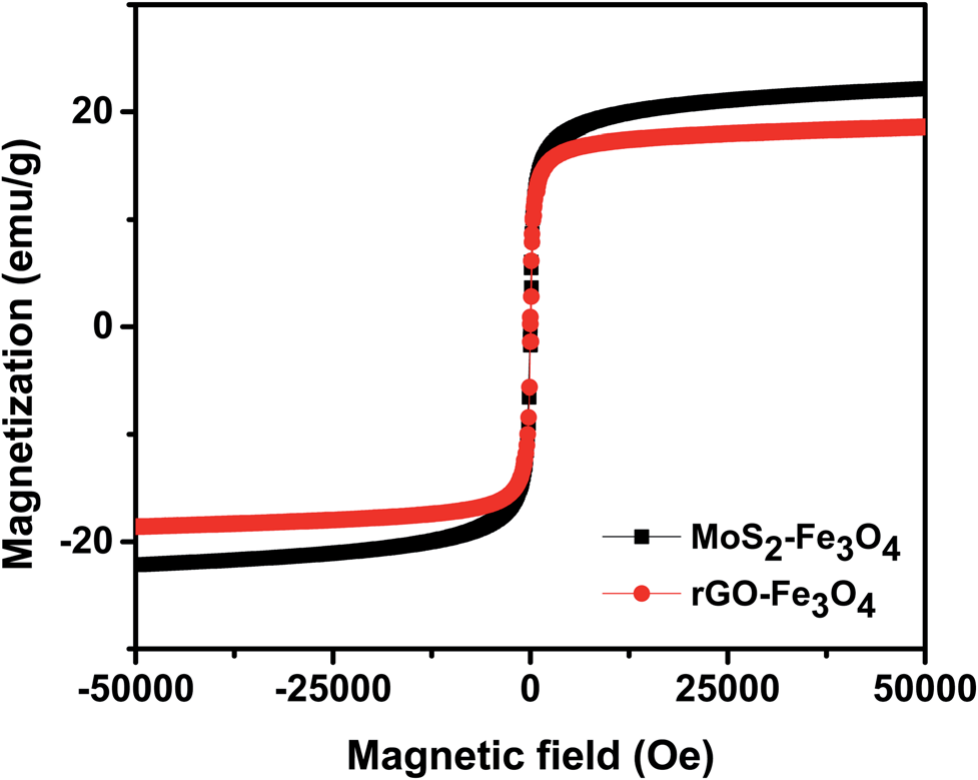
VSM plot of the synthesized nanoparticles.

Fig. 4 shows the room temperature magnetization plot of the synthesized nanoparticles. rGO–Fe_3_O_4_ shows a slightly lower saturation magnetization of 18.6 emu g^−1^ than MoS_2_–Fe_3_O_4_, which shows a saturation magnetization of 22.2 emu g^−1^. This is in accordance with the EDS result, where Fe atomic weight% was found to be higher in MoS_2_–Fe_3_O_4_ as compared to rGO–Fe_3_O_4_.

### 4.2 Morphological analysis of the multilayered assembly

**Fig. 5:**
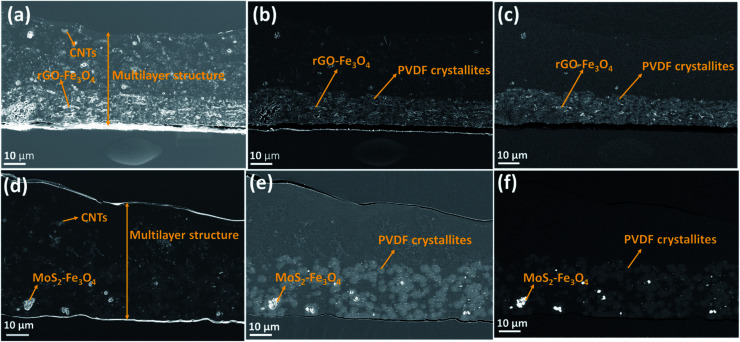
SEM micrographs of the multilayered film's cross-section (embedded in epoxy and cut using an ultramicrotome). (a–c) PVDF(rGO–Fe_3_O_4_)/M/PC (d–f): PVDF(MoS_2_–Fe_3_O_4_)/M/PC. Each row depicts the same area with different detectors: in-lens, SE2, and back-scattered electrons detectors, respectively.

**Fig. 6:**
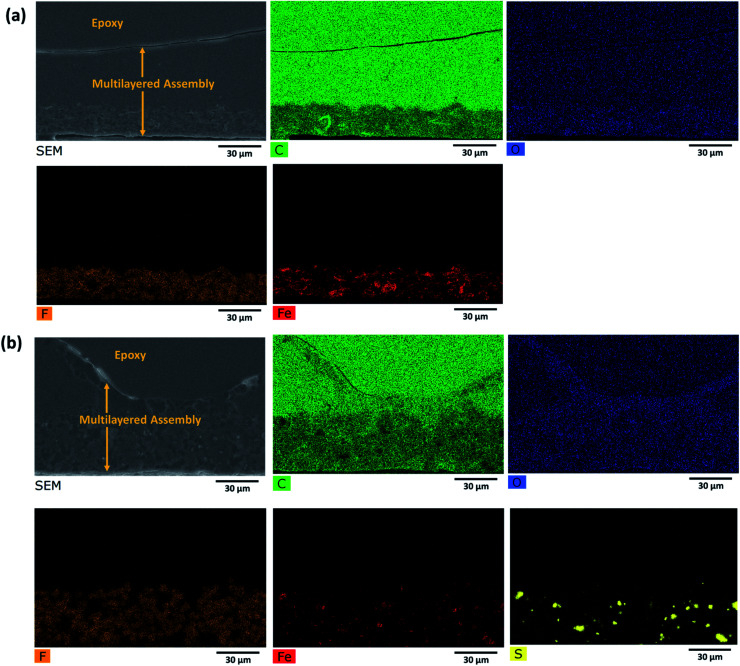
EDS map of the cross section of the multilayered film: (a) PVDF(rGO–Fe_3_O_4_)/M/PC; (b) PVDF(MoS_2_–Fe_3_O_4_)/M/PC.

**Fig. 7:**
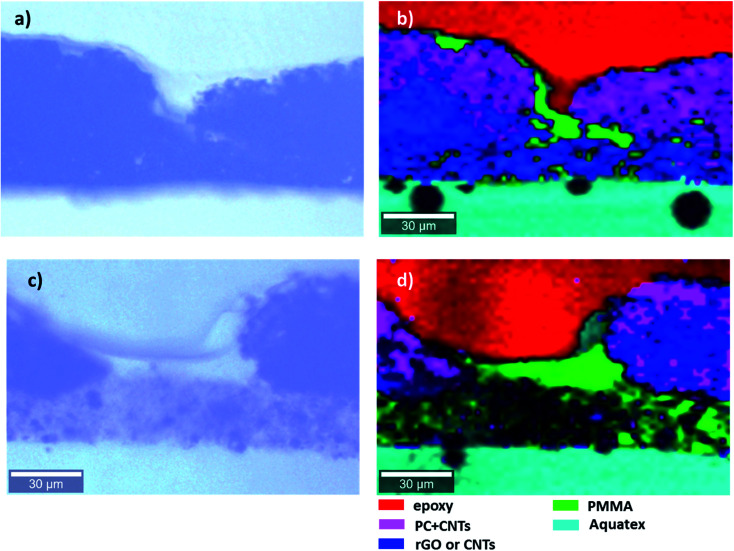
Light microscopy images (a and c) and Raman map (b and d) of the cross section of the multilayered film: (a and b) PVDF(rGO–Fe_3_O_4_)/M/PC; (c and d) PVDF(MoS_2_–Fe_3_O_4_)/M/PC.

Such a complex multilayer system could only be characterized by two complementary methods, SEM and Raman microscopy, using the strengths of each of the methods to clarify various aspects of the assembly. SEM imaged the CNTs (with the in-lens detector, Fig. 5a and d) and Fe_3_O_4_ nanoparticles (with a back-scattered electrons detector, Fig. 5c and f). PVDF gives sufficient contrast in back-scattered electrons with respect to PMMA and PC, but PMMA cannot be distinguished from PC. EDS (Fig. 6) was further used to confirm the location of MoS_2_, Fe_3_O_4,_ and PVDF. Unfortunately, EDS cannot distinguish PMMA from PC either. On the other hand, PMMA can be distinguished easily from PC with Raman microscopy (Fig. 7). However, due to the spatial resolution of 300 nm, Raman microscopy cannot detect MoS_2_ and Fe_3_O_4_ nanoparticles.

Fig. 5a–c show the SEM micrographs of the multilayered film's cross-section containing rGO–Fe_3_O_4_, while Fig. 5d–f show the SEM micrographs of the cross-section of the multilayered assembly containing MoS_2_–Fe_3_O_4_. In-lens, SE2 (type II secondary electron), and back-scattered electron detectors are three different SEM detectors used to obtain the images for the same location. The micrographs in Fig. 5a and d are obtained by the in-lens detector (generally surface sensitive and used for high-resolution micrographs; in this case, gives the best possible contrast of CNTs), Fig. 5b and e show the results obtained by the SE2 detector (generally for imaging the topography; in this case for imaging both topography and material contrast) and Fig. 5c and f show the results obtained by the back-scattered electron detector (for enhanced material contrast or atomic number contrast).

At first sight, the assembly consists of two layers: a uniform lower layer and an upper layer with varying thickness. CNTs are visible as white dots, mostly in the upper layer of PC; however, there seems to be a slight diffusion across the layers, as evident from Fig. 5a and d. PVDF is present in the lower layer – the light grey areas in Fig. 5b, c, e and f. In the nanocomposite with rGO–Fe_3_O_4_, PVDF makes most of the lower layer. In the nanocomposite with MoS_2_–Fe_3_O_4_, PVDF forms spheres uniformly distributed in another polymer. The PVDF is unanimously identified by the presence of fluorine, as can be seen in EDS mapping (F map) in Fig. 6 and S6.† The SEM micrographs show that the hybrid nanoparticles (rGO–Fe_3_O_4_ or MoS_2_–Fe_3_O_4_) are mostly restricted to the PVDF layer. Additionally, EDS confirms the location of Fe_3_O_4_ (Fe map) and MoS_2_ (S map), which were attributed to the white dot areas in Fig. 5c and f. Fe and S mapping also confirm that the PVDF portion's layer incorporates maximum Fe_3_O_4_ and MoS_2_–Fe_3_O_4_ as expected. Carbon and oxygen EDS mappings cannot be used to distinguish epoxy, PMMA, and PC. Also, it should be noted that the SEM micrographs show no sharp distinguishable interface between the three polymers, suggesting the diffusion of polymers across the interface with no slippage of individual layers (no gaps or cracks between the three different polymeric layers).

Raman microscopy reveals the location of epoxy (red), PMMA (green), and PC (pink) in Fig. 7. PMMA is present with PVDF and PC, suggesting the diffusion of PMMA in both directions as an interfacial agent, sticking the layers of two incompatible polymers (PVDF and PC). This might be due to a combination of factors such as the partial miscibility of PVDF/PMMA and PMMA/PC in the solution state, solvent selection, and the drying time allotted for each layer of the stack processing. PC is not present as a pure component; it always contains CNTs (black areas in light microscopy). In these particular measurements, CNTs were not distinguishable from rGO, and the blue color represents either of the components in Fig. 7. However, CNTs and rGO are well distinguishable in the SEM micrographs. The cyan color marks the presence of an Aquatex embedding substance for light microscopy.

From Fig. 5, we can also observe the variation in thickness along the cross-section of the multilayered film. PVDF, along with hybrid nanoparticles, was cast first, and it faced the glass surface. Hence the PVDF side is comparatively flatter than the PC side, which was exposed to air. This might be because of the difference in the solvent's evaporation rates or the not-so-well dispersed CNTs forming agglomerates, which manifested as bumps in the upper layer. The average thickness of the multilayered assembly is observed to be 53 μm. Also, we observe that this technique has the added advantage of achieving continuous layers of polymeric films with no sharp interface, thus avoiding any slippage between individual layers. Thus, PMMA interfacially locks the PC and PVDF layers.

### 4.3 Electrical conductivity in the multilayered assembly

**Fig. 8:**
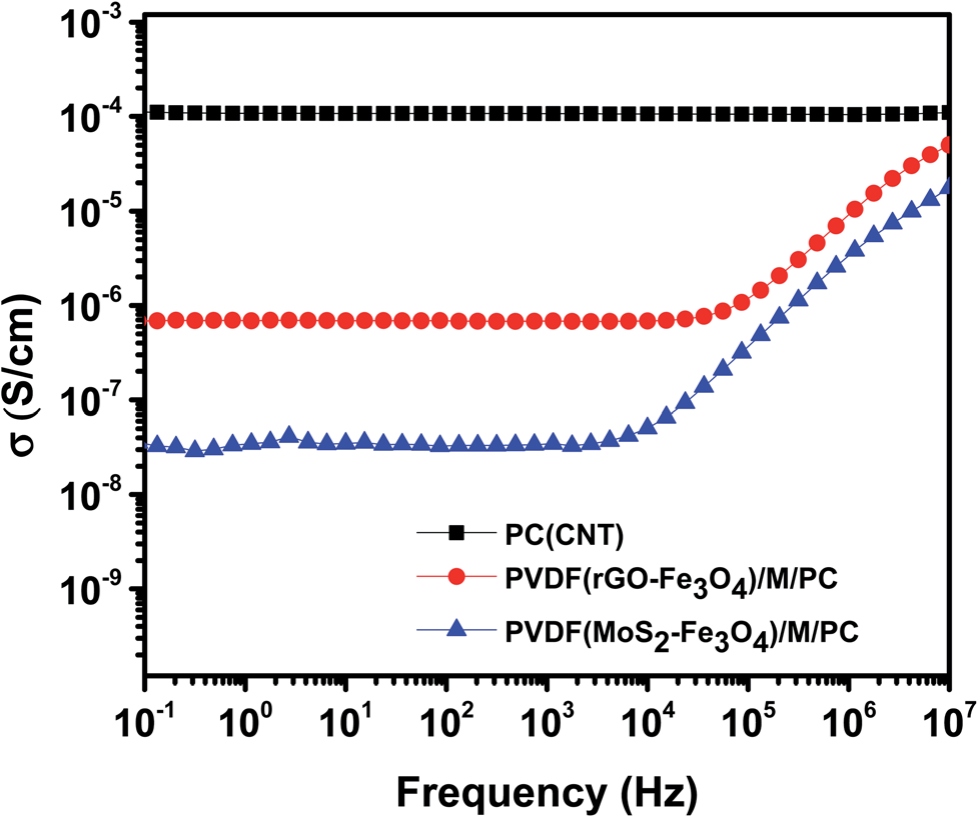
AC electrical conductivity as a function of frequency.

Fig. 8 shows the AC electrical conductivity measurements of multilayered assemblies of PVDF(rGO–Fe_3_O_4_)/M/PC and PVDF(MoS_2_–Fe_3_O_4_)/M/PC with respect to the control film of single layer PC (CNT). The ac electrical conductivity was measured as a function of frequency at room temperature. PC, PMMA, and PVDF are insulating polymers. Multiwalled carbon nanotubes are known to be intrinsically conductive in nature, with an electrical conductivity value of 10^6^ to 10^7^ S m^−1^.^12^ Hence the conductivity of PC (CNT) is the highest as compared to the multilayered assemblies. The conductivity of rGO is controlled by reducing the functional groups, and it is observed to be lower than that of CNTs.^41^ Fe_3_O_4_ and MoS_2_ are known to be semiconducting. Fe_3_O_4_ has a bandgap of 2.51–3.01 eV,^13^ while MoS_2_ has a bandgap of 1.23 eV.^14^ Considering the multilayered assembly, the ac electrical conductivity showed a frequency-independent plateau at a lower frequency following the universal power-law fitting as per eqn (1).^39,42^

1 *σ*′(*ω*) = *σ*(0) + *σ*_AC_(*ω*) = *σ*_DC_ + *Aω*^*s*^

Here, the exponent “*s*” ranges from 0 to 1 and depends on the temperature and frequency. It indicates the extent of charge transfer that occurs through tunneling/hopping. It denotes the degree of connectedness of long-range charge hopping pathways or the extent of tortuosity for mobile charges.^43^

Here, it is to be noted that the CNTs were concentrated in the insulating PC layer, and thus this layer is expected to be highly conducting in nature. PMMA and PVDF are insulating polymers, and rGO–Fe_3_O_4_ and MoS_2_–Fe_3_O_4_ are semiconducting fillers. Hence the mid-layer and bottom layer should have ideally made the entire multilayered assembly insulating in nature. But this was not the case, as shown in Fig. 8. This implies that some CNTs have diffused through the layers, as observed in the SEM micrographs, where no sharp and distinct interface at the layer boundary was found.

PVDF(MoS_2_–Fe_3_O_4_)/M/PC shows a lower dc electrical conductivity of 3.6 × 10^−8^ S cm^−1^ compared to PVDF(rGO–Fe_3_O_4_)/M/PC, which shows a dc plateau at 6.8 × 10^−7^ S cm^−1^ at 10^−1^ Hz. This can be attributed to MoS_2_, which has a bandgap of 1.23 eV. MoS_2_ might be comparatively more semiconducting (higher bandgap) than rGO, thus reflecting the difference in the dc plateau value. Also, there might be a possibility that the sheet-like larger structure of rGO makes the percolation threshold lower as compared to that of flower-like MoS_2_. By fitting the power law, “*s*” is observed to be 0.99 for PVDF(MoS_2_–Fe_3_O_4_)/M/PC and 0.89 for PVDF(rGO–Fe_3_O_4_)/M/PC indicating that charge transfer occurs *via* tunneling/hopping. This nature of the curve is typical to resistor–capacitor (R–C) networks, representing a microstructure that contains both dielectric (the capacitor) and conductive regions (the resistor).^44^ The fitting suggests that as one moves from MoS_2_–Fe_3_O_4_ to rGO–Fe_3_O_4_ as a filler, the capacitor content is found to decrease from 99% to 89%.

However, the single-layered PC(CNT) exhibits frequency-independent dc conductivity in the entire measured range. This indicates that the PC(CNT) film consists of well-connected CNT pathways. The resistor aspect of the network is far more dominant than the capacitance aspect, which is considered negligible. And the dominant mechanism of charge transfer is *via* tunneling. Additionally, the real permittivity plot of PC(CNT), PVDF(rGO–Fe_3_O_4_)/M/PC, and PVDF(MoS_2_–Fe_3_O_4_)/M/PC is shown in Fig. S7.†

### 4.4 Melt rheological response of the individual composite layers and mechanical characterization of the multilayered assembly

Rheology aims at studying the deformation and flow behavior of materials. Rheological measurements give an idea about the filler–filler network, filler–polymer interaction, and the state of dispersion. Polymers and polymer composites show a viscoelastic response to the applied shear.

**Fig. 9:**
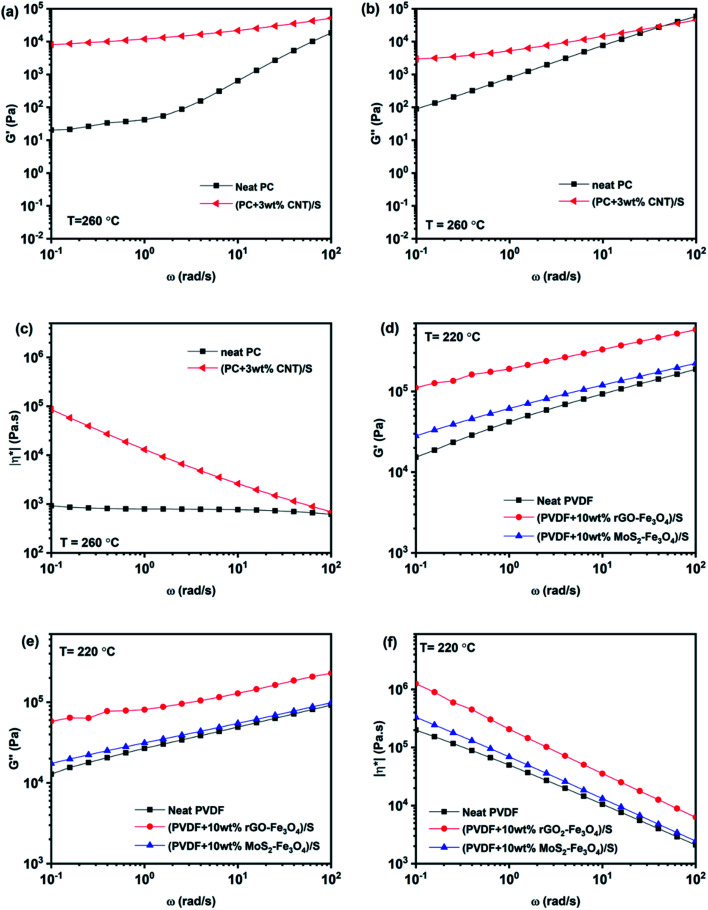
Melt rheological measurements: storage modulus (a and d), loss modulus (b and e), and complex viscosity (c and f) *vs.* angular frequency plots of PC and PVDF based composites.

To understand the individual layers' rheological properties, PC + 3 wt% CNT, PVDF + 10 wt% rGO–Fe_3_O_4,_ and PVDF + 10 wt% MoS_2_–Fe_3_O_4_ solution mixed samples were prepared and compression-molded at 260 °C, 220 °C and 220 °C, respectively, to form individual disks of 25 mm diameter and 1 mm thickness. PC + 3 wt% CNT, PVDF + 10 wt% rGO–Fe_3_O_4_ and PVDF + 10 wt% MoS_2_–Fe_3_O_4_ solution mixed-compression molded disc samples are represented as (PC + 3 wt% CNT)/S, (PVDF + 10 wt% rGO–Fe_3_O_4_)/S, and (PVDF + 10 wt% MoS_2_–Fe_3_O_4_)/S respectively. The melt rheological measurements of PC-based composites were carried out at 260 °C, and PVDF-based composites were tested at 220 °C using a parallel plate setup. The second sweep results in the frequency range of 100 rad s^−1^ to 0.1 rad s^−1^ are compiled in Fig. 9.

Frequency sweep describes the time-dependent response of the sample in a non-destructive deformation range. High frequencies represent fast motion on short timescales, whereas low frequencies simulate slow motion on long timescales or rest. The amplitude is kept constant, while the oscillation frequency is decreased step-wise from one measuring point to the next. With an increase in the angular frequency, *G*′ and *G*′′ increase, while complex viscosity decreases, as shown in Fig. 9. As observed in Fig. 9c, (PC + 3 wt% CNT)/S is orders of magnitude more viscous than neat PC, and this behavior is more pronounced at low frequencies. The reduction in |η*| with angular frequency in (PC + 3 wt% CNT)/S is a typical behavior exhibited by shear-thinning materials. It is also to be noted that neat PC shows negligible frequency dependence, and the complex viscosity increases with the CNT content, which is in accordance with the existing literature.^45^ The increase in complex viscosity with the addition of CNTs is primarily due to the increase in storage modulus *G*′, as is seen in Fig. 9a. As compared to *G*′, the increase in the values of *G*′′ is lower. It is observed that the slope of the storage modulus in the terminal region decreases with the addition of CNTs, which conforms to the literature.^46^ The higher modulus and solid-like plateau behavior at low frequencies can be attributed to the active interactions between CNTs. It indicates that the percolated CNTs led to developing a solid-like network between PC and CNTs.^47^ In (PC + 3 wt% CNT)/S, *G*′ is observed to be higher than *G*′′, indicating that the system exhibits a gel-like response with percolation being prominent in the nanocomposite.^46^

Similarly, neat PVDF, (PVDF + 10 wt% rGO–Fe_3_O_4_)/S, and (PVDF + 10 wt% MoS_2_–Fe_3_O_4_)/S also show shear thinning behavior. However, the enhancement in the rheological parameters (*G*′, *G*′′ and |η*|) by filler addition is small in the case of (PVDF + 10 wt% MoS_2_–Fe_3_O_4_)/S, while it is more prominent in the case of (PVDF + 10 wt% rGO–Fe_3_O_4_)/S. This can be attributed to the better interaction due to the large sheet-like structure of rGO compared to the structure of MoS_2_, which is also confirmed from the SEM images shown in Fig. 5. A sheet-like structure of rGO holds the ability to lower the percolation threshold and enhance the polymer–filler interaction due to the large interfacial area. It is to be noted that the complex viscosity in neat PVDF exhibits a frequency dependent response, unlike neat PC, which is in accordance with the existing literature.^48^

**Fig. 10:**
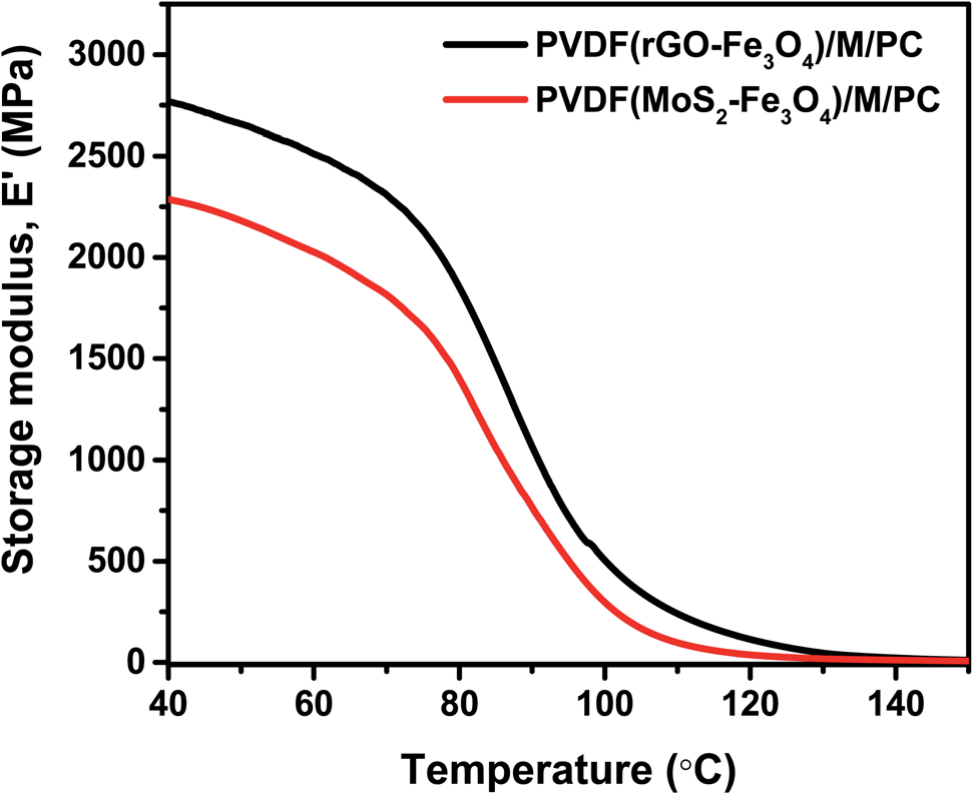
DMA plot of the storage modulus as a function of temperature.

As explained by rheology, polymers/polymer composites are viscoelastic entities; hence they exhibit an in-phase and out-of-phase response to the applied input strain. To study the viscoelastic response of the multilayered assembly, DMA was performed in tension mode. Thus, the multilayered assembly's mechanical behavior was investigated by applying a sinusoidal tensile deformation to a sample of known geometry and measuring the response. Fig. 10 shows the storage modulus, *E*′ of PVDF(rGO–Fe_3_O_4_)/M/PC, and PVDF(MoS_2_–Fe_3_O_4_)/M/PC as a function of measuring temperature. At a constant frequency and strain amplitude, the storage modulus at 40 °C for PVDF(rGO–Fe_3_O_4_)/M/PC and PVDF(MoS_2_–Fe_3_O_4_)/M/PC was found to be 2767 MPa and 2287 MPa, respectively. The storage modulus is larger in PVDF(rGO–Fe_3_O_4_)/M/PC than PVDF(MoS_2_–Fe_3_O_4_)/M/PC, which can be attributed to the better connectivity and compatibility of the multilayered assembly with rGO due to its large surface area, as compared to MoS_2_. This is also in accordance with the rheology analysis, where (PVDF + 10 wt% rGO–Fe_3_O_4_)/S proved to have a higher storage modulus than (PVDF + 10 wt% MoS_2_–Fe_3_O_4_)/S. Thin films of PVDF(rGO–Fe_3_O_4_) and PVDF(MoS_2_–Fe_3_O_4_) fabricated using the doctor blade method had cracks and wear and hence not suitable for mechanical analysis (shown in Fig. S5†).

On the other hand, the PC(CNT) film did not show any visible wear, but it was structurally inferior for DMA analysis. It is to be noted that PC is a brittle polymer, but due to the flexibility and high aspect ratio of CNTs, the incorporation of CNTs in PC is possible. However, the low thickness of the PC(CNT) film is probably the reason for its structural inferiority. It is worth noting that incorporating nanofillers such as rGO–Fe_3_O_4_ and MoS_2_–Fe_3_O_4_ in PC film casting, especially at such low thicknesses, may not be feasible (extreme brittleness). This is the reason why CNTs were incorporated in PC, and the dielectrically lossy nanofiller (rGO–Fe_3_O_4_ or MoS_2_–Fe_3_O_4_) was incorporated in PVDF with an interfacial lock using PMMA. Fig. S5† also suggests that a single layer of the PVDF composite film or PC composite film with abundant nanofillers resulted in inferior film quality compared to multilayered films at a similar film thickness. We can conclude that our approach to shifting from single-layered composites to multilayered films is worthful, seeing the mechanical property enhancement in the multilayered assembly compared to the single-layered composite film.

As a control, one can consider comparing the multilayered composite film's storage modulus with the neat commercial polymers’. The storage modulus of the compression-molded samples of commercial PC (same grade) and PVDF (same grade) is reported to be 2440 MPa and ∼500 MPa, respectively, at 40 °C.^49,50^

### 4.5 Microwave shielding ability of the multilayered stack

The total shielding effectiveness, SE_T,_ is defined in terms of the logarithm of the ratio of the incident power (*P*_I_) to the transmitted power (*P*_T_) through the shield material.^39^2
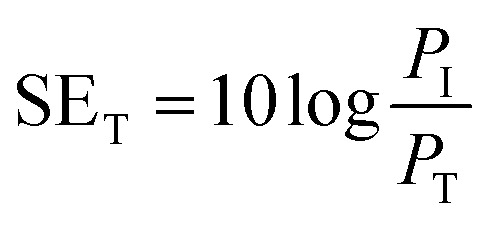


SE_T_ is the shield's ability to attenuate EM radiation, and it is expressed in units of dB. Shielding occurs *via* three mechanisms, namely shielding *via* reflection (SE_R_), shielding *via* absorption (SE_A_), and shielding *via* multiple reflections (SE_MR_).

3 SE_T_ = SE_A_ + SE_R_ + SE_MR_

when SE_T_ >15 dB or when the shield thickness is greater than the skin depth, SE_MR_ is ignored, and thus SE_T_ can then be expressed as

4 SE_T_ = SE_A_ + SE_R_

SE_A_, SE_R_ and SE_MR_ are theoretically expressed according to the following equations:5
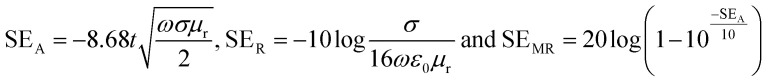
where *σ* is the total conductivity, *ω* is the angular frequency (*ω* = 2π*f*), *μ*_r_ corresponds to the relative permeability of the shield material, *t* is the thickness of the shield, and *ε*_0_ represents the dielectric constant in free space.

A two-port VNA is used to measure the shielding effectiveness experimentally. Since it is difficult to measure the current and voltage at such a high frequency precisely, the instrument measures the scattering parameters (*S*_11_, *S*_12_, *S*_21,_ and *S*_22_), which in turn is used to calculate SE_T_, SE_A_ and SE_R_ using the following equations:6
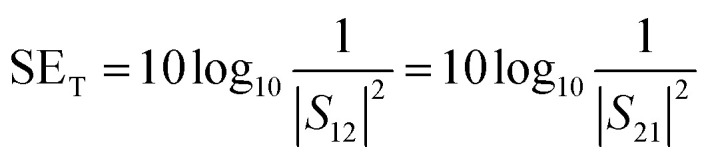
7
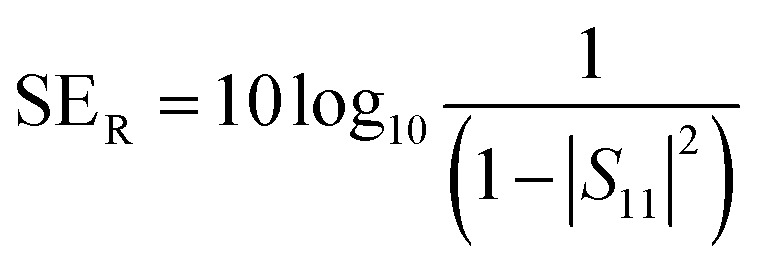
8
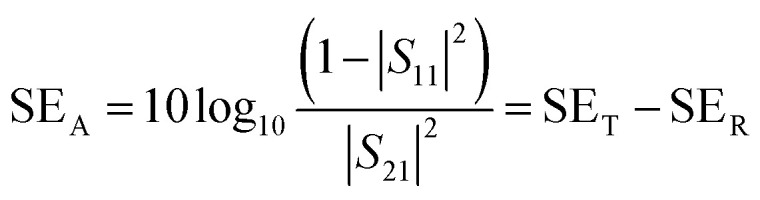
where *S*_11_, and *S*_22_ represent the reflection coefficient and *S*_12_, and *S*_21_ represent the absorption coefficient.

**Fig. 11:**
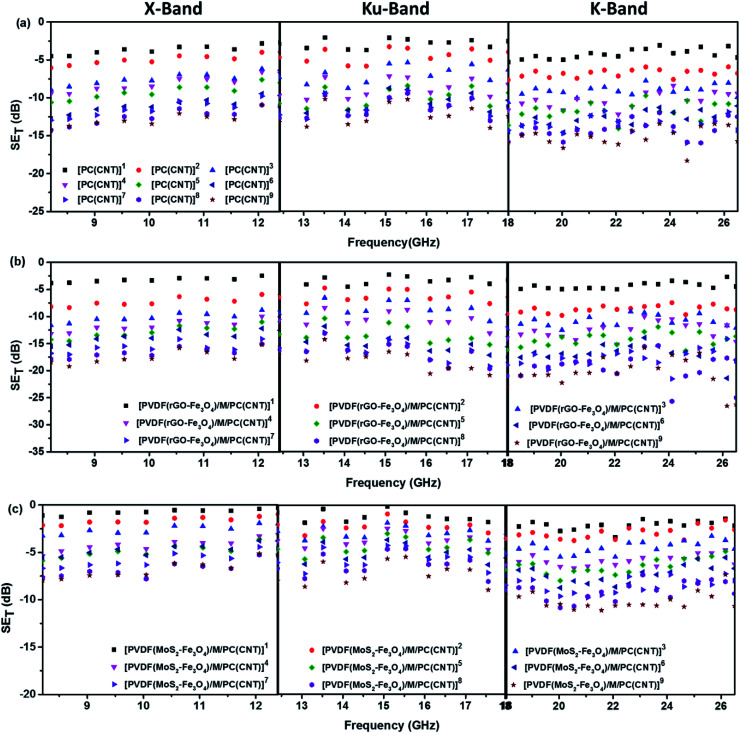
SE_T_*vs.* frequency for (a) PC(CNT)- set of 9 stacks in an ascending order, (b) PVDF(rGO–Fe_3_O_4_)/M/PC – set of 9 stacks in an ascending order and (c) PVDF(MoS_2_–Fe_3_O_4_)/M/PC – set of 9 stacks in an ascending order. Note that the legend is common for the plots in one row, *i.e.*, for one type of sample across different frequency bands.

**Scheme 4:**
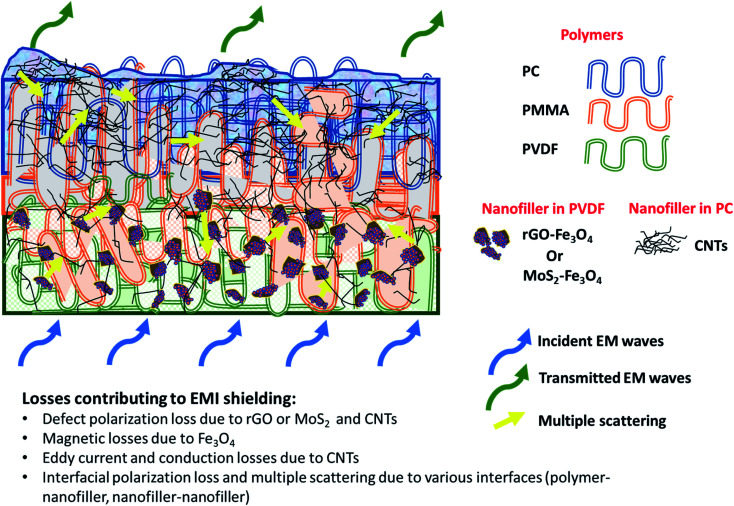
Loss mechanism involved in the multilayered assembly.

Fig. 11 shows SE_T_ as a function of frequency in the X (8.2–12.4 GHz), Ku (12.4–18 GHz), and K (18–26.5 GHz) bands. The SE_T_ values of three different samples, namely PC(CNT), PVDF(rGO–Fe_3_O_4_)/M/PC, and PVDF(MoS_2_–Fe_3_O_4_)/M/PC, are compiled and presented. The films were sequentially stacked one above the other using an acrylic-based adhesive, and the number of stacked layers is denoted by ‘*z*’. For example, [PVDF(nanofiller name)/M/PC]^1^ denotes a multilayered film in which shielding is measured by stacking one multilayered film, [PVDF(nanofiller name)/M/PC]^2^ denotes a multilayered film where shielding is measured by stacking two multilayered films; [PC(CNT)]^9^ denotes single-layered PC(CNT) film where shielding is measured by stacking nine such films, and so on. It is observed that [PVDF(rGO–Fe_3_O_4_)/M/PC]^9^ shows a SE_T_ of −26.3 dB, [PVDF(MoS_2_–Fe_3_O_4_)/M/PC]^9^ shows a SE_T_ of −10.6 dB and [PC(CNT)]^9^ shows a SE_T_ of −15.7 dB at 26.5 GHz frequency. Thus, rGO–Fe_3_O_4_ proved to be a better filler than MoS_2_–Fe_3_O_4_ to maximize the SE_T_ value. The losses in PVDF(rGO–Fe_3_O_4_)/M/PC can be attributed to the multilayered assembly's adequate conductivity, the dielectric and magnetic losses due to rGO–Fe_3_O_4,_ and the multiple scattering phenomena. Multiple scattering arises due to the multiple interfaces created by the nanofiller–polymer and nanofiller (type 1)–nanofiller (type 2), as shown in Scheme 4. The shielding in PC(CNT) is mainly due to the high conductivity of the layer owing to the presence of interconnected CNTs, as is obvious from Fig. 8. However, PVDF(MoS_2_–Fe_3_O_4_)/M/PC showed the least SE_T_, probably because of the film's low conductivity, which arose from the semiconducting MoS_2_ obstructing the pathway for charge transfer. Also, rGO is a sheet-like larger structure, compared to flower-like MoS_2_. This can be another possible cause of PVDF(rGO–Fe_3_O_4_)/M/PC exhibiting a higher conductivity (lower percolation threshold) as compared to PVDF(MoS_2_–Fe_3_O_4_)/M/PC. It is to be noted that conducting fillers (such as CNTs and to some extent rGO) give rise to conduction losses and eddy current losses, which adds to shielding performance. Also, it is observed that the saturation magnetization of MoS_2_–Fe_3_O_4_ is higher. Still, it did not prove to be a superior filler in the desired frequency range, thus confirming that magnetic properties may not have a significant impact on shielding in the chosen frequency range, which is as expected from Snoek's law. Besides, the flower-like morphology of MoS_2_ creates additional interfaces, which could have added to the shielding performance through interfacial polarization and multiple scattering. However, the poor shielding performance of PVDF(MoS_2_–Fe_3_O_4_)/M/PC only suggests that shielding efficiency is a trade-off between multiple factors. The dielectric losses expected from MoS_2_, the interfacial losses, and multiple scattering arising from the flower-like morphology of MoS_2_ as well as other interfaces are not enough to make PVDF(MoS_2_–Fe_3_O_4_)/M/PC suitable for commercial shielding applications (∼−20 dB). This also suggests that the choice of fillers and distribution of fillers significantly affect the shielding performance. It is to be noted that the average thickness of the multilayered assembly (for 1 layer) is found to be 53 μm as obtained from the SEM micrograph (shown in Fig. 5). The shielding effectiveness results are also compiled for PVDF(rGO–Fe_3_O_4_) and PVDF(MoS_2_–Fe_3_O_4_), and they were found to be −0.85 dB and −0.01 dB at 26.5 GHz, respectively. This confirms that semiconducting/magnetic fillers can generally not show high SE_T_ only by themselves (Fig. S8†). It is to be noted that the thickness of the EMI shielding specimens is similar and hence comparable. As a control experiment, 1 mm disc of PC + 3 wt% CNT + 10 wt% rGO–Fe_3_O_4_ was prepared by melt mixing, followed by the compression molding technique, and SE_T_ was found to be approximately −20 dB at 26.5 GHz frequency, which is significantly lower than that of the multilayered stack of 9 layers (SE_T_ = −26.3 dB @ 480 μm). The melt mixing-compression molding approach was chosen as a control experiment because a single layered doctor-blade casting with a high nanofiller content gave a structurally inferior film, as already mentioned in Section 4.4. Even the melt mixing approach, with a high nanofiller content, results in processing difficulty, suggesting that a multilayered doctor-blade casting approach can be considered a competent alternative to incorporate a high nanofiller concentration at low thicknesses. The addition of PMMA on its own might not result in an enhanced shielding performance, but this approach allows the addition of a high content of nanofillers at a low thickness (indirectly contributing to enhanced shielding) while maintaining the mechanical performance.

**Fig. 12:**
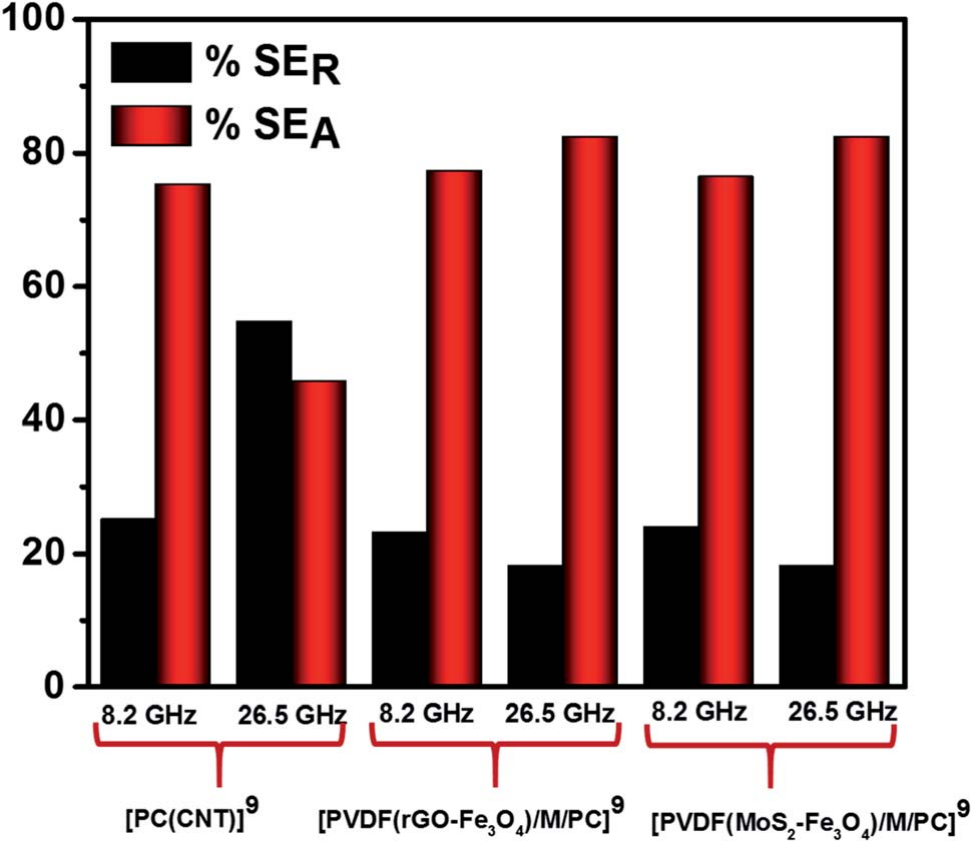
SE_A_ and SE_R_ for [PC(CNT)]^9^, [PVDF(rGO–Fe_3_O_4_)/M/PC]^9^ and [PVDF(MoS_2_–Fe_3_O_4_)/M/PC]^9^ at 8.2 and 26.5 GHz frequency.

Fig. 12 illustrates the percentage of absorption/reflection for [PC(CNT)]^9^, [PVDF(rGO–Fe_3_O_4_)/M/PC]^9^, and [PVDF(MoS_2_–Fe_3_O_4_)/M/PC]^9^ at 8.2 and 26.5 GHz frequency. The absorption/reflection plot for one stack is shown in Fig. S9.† The percentage of absorption for [PC(CNT)]^9^ is found to be 45.5, for [PVDF(rGO–Fe_3_O_4_)/M/PC]^9^ is found to be 82.1, and for [PVDF(MoS_2_–Fe_3_O_4_)/M/PC]^9^ is found to be 82.1 at 26.5 GHz frequency. For the multilayered assemblies of PVDF(rGO–Fe_3_O_4_)/M/PC and PVDF(MoS_2_–Fe_3_O_4_)/M/PC, absorption is the dominant shielding mechanism, irrespective of the frequency in consideration. This can be attributed to the losses from defect dipole polarization of MoS_2_, defect dipole polarization of rGO, magnetic losses from Fe_3_O_4_, multiple interfacial polarizations from MoS_2_/Fe_3_O_4_, and rGO/Fe_3_O_4_ hybrids, and the CNT/nanoparticle–polymer interface.^15^ The loss mechanism is summarized in Scheme 4. In the case of PC(CNT), the reflection percentage fluctuates drastically with thickness as well as frequency, and it is rather difficult to find a correlation based on current data.

## 5. Conclusions

A novel approach was developed to fabricate a multilayered assembly of polymeric thin-film composites to enhance EMI shielding properties while maintaining structural stability. rGO–Fe_3_O_4_ and MoS_2_–Fe_3_O_4_ were synthesized using hydrothermal synthesis, and the doctor blade setup was used to sequentially stack PVDF, PMMA, and PC layers along with fillers (rGO–Fe_3_O_4_ or MoS_2_–Fe_3_O_4_ (10 wt%) in PVDF and CNTs (3 wt%) in PC). The fabrication approach was chosen such that PMMA acts as a sandwich layer to enhance the interfacial adhesion between the PC/PVDF system. The multilayered assemblies of PVDF–rGO–Fe_3_O_4_/PMMA/PC–CNT (or PVDF(rGO–Fe_3_O_4_)/M/PC) and PVDF–MoS_2_-Fe_3_O_4_/PMMA/PC–CNT (or PVDF(MoS_2_–Fe_3_O_4_)/M/PC) were tested for EMI shielding performance in the broadband frequency range (8.2–26.5 GHz), along with mechanical properties.

The SEM micrographs and Raman mapping showed no sharp interface between the layers, proving that PMMA diffused and acted as a stitching layer to lock the PVDF and PC composite layers interfacially. The average thickness of the multilayer film was observed to be ∼53 μm. The mechanical properties of PVDF(rGO–Fe_3_O_4_)/M/PC and PVDF(MoS_2_–Fe_3_O_4_)/M/PC were measured using DMA. At a constant frequency and strain amplitude, the storage modulus at 40 °C for PVDF(rGO–Fe_3_O_4_)/M/PC and PVDF(MoS_2_–Fe_3_O_4_)/M/PC was found to 2767 MPa and 2287 MPa, respectively. This value is comparable/higher than the storage modulus of compression-molded samples of neat PC and PVDF, which is reported to be 2440 MPa and ∼500 MPa respectively at 40 °C^49,50^

By stacking nine multilayered films (denoted by a superscript in nomenclature) one above the other, reaching an assembly thickness of *ca.* 480 μm, the total EMI shielding effectiveness (SE_T_) at 26.5 GHz frequency was found to be −26.3 dB and −10.6 dB for [PVDF(rGO–Fe_3_O_4_)/M/PC]^9^ and [PVDF(MoS_2_–Fe_3_O_4_)/M/PC]^9^, respectively. This is significantly higher than the SE_T_ value of a 1 mm disc of PC + 3 wt% CNT + 10 wt% rGO–Fe_3_O_4_, obtained from a melt mixing, followed by a compression molding approach (approx. −20 dB for 1 mm thickness). Also, rGO–Fe_3_O_4_ proved to be a better filler than MoS_2_–Fe_3_O_4_ in enhancing mechanical stability and improving SE_T_. Shielding *via* absorption is the dominating mechanism for multilayered films, irrespective of the number of stacked layers and the frequency under consideration (8.2–26.5 GHz). The loss mechanism in PVDF(rGO–Fe_3_O_4_)/M/PC can be attributed to the multilayered assembly's adequate conductivity, the dielectric and magnetic losses due to rGO–Fe_3_O_4,_ and the multiple scattering phenomena. Though [PC(CNT)]^9^ showed a SE_T_ of −15.7 dB at 26.5 GHz frequency, it was primarily reflection-based shielding, which can be attributed to the high conductivity induced by the presence of the CNT network. However, PVDF(MoS_2_–Fe_3_O_4_)/M/PC showed the lowest SE_T_, probably because of this film's lower conductivity, which arose from the semiconducting nature of MoS_2_ obstructing the pathway for charge transfer. Despite the dielectric properties of MoS_2_ and higher saturation magnetization of MoS_2_–Fe_3_O_4_, it showed a lower shielding performance. This suggests that magnetic materials work more effectively in the lower frequency range, as is also proposed by Snoek's limit. Also, large sheet-like rGO in PVDF(rGO–Fe_3_O_4_)/M/PC made the percolation threshold lower than that of the flower-like MoS_2_ in PVDF(MoS_2_–Fe_3_O_4_)/M/PC. Our study also deduces that the multilayered strategy can lead to a higher SE_T_ value as observed when incorporated with the suitable nanofiller.

To the best of our knowledge, this is the first time in the literature that polymer layering has been performed sequentially using the doctor blade setup in EMI shielding applications. Moreover, polymer and polymer–solvent pair selection is unique with respect to their type, and this technique has proven to be efficient in enhancing the SE_T_ value while maintaining mechanical stability. Besides, this technique may improve the SE_T_ value, but the generalization is somewhat tricky as the SE_T_ value is a trade-off between several parameters. But the appropriate choice of fillers and tuning the filler quantity and distribution might help enhance the shielding efficiency while maintaining the structural properties.

## Conflicts of interest

There are no conflicts to declare.

## Supplementary Material

NA-003-D0NA01071E-s001
