# Supplementary material for: Ultrathin structures derived from interfacially modified polymeric nanocomposites to curb electromagnetic pollution
Source: Nanoscale Adv. 2021 Mar 8;3(9):2632–48. doi: 10.1039/d0na01071e (PMC9419275; doi:10.1039/d0na01071e)
Supplement: NA-003-D0NA01071E-s001 [file NA-003-D0NA01071E-s001.pdf]

Electronic Supplementary Material (ESI) for Nanoscale Advances.
This journal is © The Royal Society of Chemistry 2021

Supporting Information

Ultrathin structures derived from interfacially modified polymeric nanocomposites to curb electromagnetic pollution

Kumari Sushmita [a], Petr Formanek [b], Dieter Fischer [b], Petra Pötschke [b], Giridhar Madras [c], Suryasarathi Bose [d]*

[a] Centre for Nanoscience and Engineering, Indian Institute of Science, Bangalore- 560012, India

[b] Leibniz-Institut für Polymerforschung Dresden e. V., Dresden-01069, Germany

[c] Interdisciplinary Centre for Energy Research, Indian Institute of Science, Bangalore- 560012, India

[d] Department of Materials Engineering, Indian Institute of Science, Bangalore- 560012, India

***Corresponding author**: sbose@iisc.ac.in

1) The conceptual basis for the choice of polymer and solvent

For a preliminary test of miscibility, neat films and multilayered films were cast using a doctor blade setup and observed using digital imaging, as shown in figure S1. Before casting, the individual polymeric solutions/dispersions were prepared. The solvent used for PVDF was dimethylformamide (DMF), and PMMA and PC were chloroform ($CHCl_3$). As already mentioned, PMMA is used as the interfacial layer for better stitching of the other two layers. PMMA is soluble in both $CHCl_3$ and DMF, and hence diffusion of the PMMA layer into both the phases of the multilayered assembly can be expected.

A couple of trial experiments were performed by dissolving polymers in various solvents such as dimethylformamide (DMF), tetrahydrofuran (THF), chloroform ($CHCl_3$), and N-Methyl-2-pyrrolidone (NMP). The combinations of polymer-solvents that were attempted are shown in table S1a. The ratio of solvent to polymer was decided manually based on the viscosity of the resulting solutions, which have to be optimum for the film preparation. The ones with the tick mark in table S1a denote the systems that were practically (visually) found to dissolve at least 1g of polymer in 10 ml solvent (temperature was kept 40 °C for low boiling point solvents such as

$CHCl_3$ and THF and 100 °C for high boiling point solvents such as DMF and NMP). The multilayered films cast using these solutions were visually inspected, and only a few of the combinations were found to mechanically stable. These combinations include PVDF-DMF/PMMA-DMF/PC-$CHCl_3$, PVDF-DMF/PMMA-$CHCl_3$/PC-$CHCl_3$, PVDF-DMF/PMMA-THF/PC-THF. The idea was to choose a solvent such that PMMA can diffuse towards both the PVDF and PC sides for better stitching. The final selection of the pairs, PVDF-DMF, PMMA-$CHCl_3$, and PC-$CHCl_3$, served the purpose. The multilayered film obtained using these pairs is shown in figure S1f.

Thus, finally, the PVDF solution was prepared by dissolving 2 g PVDF in 10 ml DMF, the PMMA solution was prepared by dissolving 3 g PMMA in 10 ml $CHCl_3$, and the PC solution was prepared by dissolving 2.5 g PC in 10 ml $CHCl_3$. The films of individual layers of PC, PMMA, and PVDF were cast using a 300 µm doctor blade, and they were sufficiently dried before immersing it in cold water to peel them off from the glass substrate. The films were further dried in a vacuum oven to evaporate the water, and the final films are shown in figures S1a, S1b, and S1c. Similarly, PVDF/ PMMA and PMMA/ PC films were sequentially cast using 200 µm and 300 µm blades, and the final films are shown in figures S1d and S1e, respectively. Figure S1f shows the final film obtained by the sequential casting of the PVDF/PMMA/PC layer using 100 µm, 200 µm, and 300 µm blade (slit size). Figure S1g and S1h show the sequential stacking of PVDF followed by PC (or PVDF/PC) and PC followed by PVDF (or PC/PVDF), respectively. Doctor blades with 200 µm and 300 µm slit sizes were used to sequentially stacking these layers. The PMMA layer was purposefully not added in these two cases (figure S1g and S1h) to justify its importance as a mid-layer.

As expected, PC and PMMA films were transparent, as seen in the digital image. PVDF was opaque in appearance. As seen in the images S1d, S1e, and S1f, the films are also transparent. However, figures S1g and S1h showing both combinations of PC and PVDF indicate opaque films. Thus, the miscibility of PMMA with both PC and PVDF has played its role in enhancing the transparency of the multilayered assembly of PVDF/PMMA/PC. The films obtained finally for PVDF/PMMA, PVDF/PC, and PC/PVDF were too brittle for mechanical analysis.

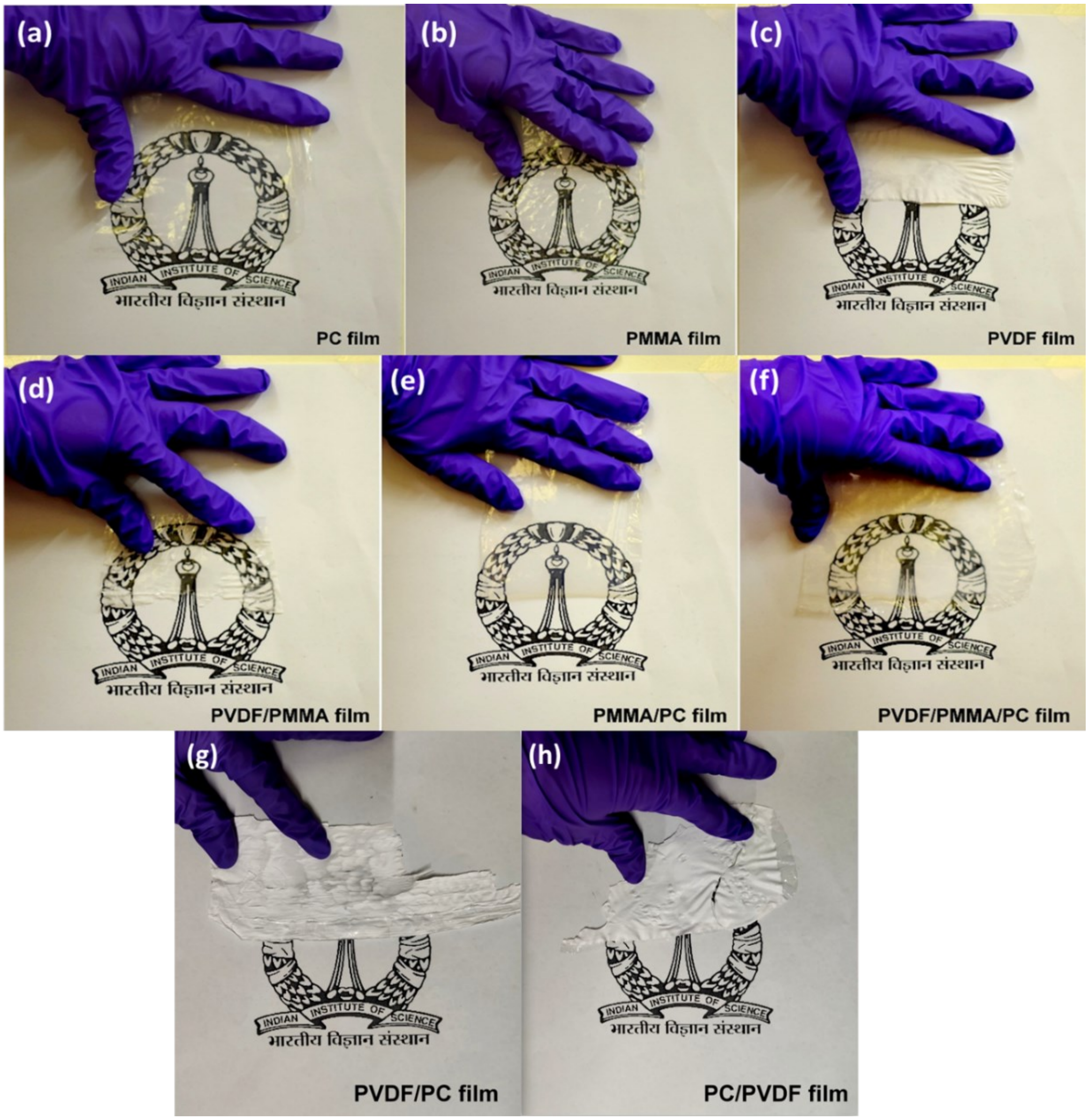

Figure S1: Digital images of (a) PC film, (b) PMMA film, (c) PVDF film, (d) PVDF/PMMA film, (e) PMMA/PC film, (f) PVDF/PMMA/PC film, (g) PVDF/PC film and (h) PC/PVDF film obtained using the doctor blade method.

Table S1b and c show the predicted polymer-solvent interactions based on the Hansen solubility parameters. Table S1b shows the value of Hansen's solubility parameters obtained from literature[1], whereas table S1c indicates if a particular solvent will dissolve the polymer or not based on equations 1 and 2.

$$R_a{}^2 = 4\,(\delta_{d2} - \delta_{d1})^2 + (\delta_{p2} - \delta_{p1})^2 + (\delta_{h2} - \delta_{h1})^2 \text{ --------------------- (1)}$$

Relative energy difference or RED = $R_{a/}\ R_0$ --------------------- (2)

where δ_d refers to the energy from dispersion forces between molecules, δ_p refers to the energy from the dipolar intermolecular force between molecules, δ_h refers to the energy from hydrogen bonds between molecules, subscript 1 and 2 stands for solvent and solute and R_0 refers to the interaction parameter of the polymer.

If

RED < 1 → polymer will dissolve

RED = 1 → polymer will partially dissolve

RED > 1 → polymer will not dissolve

The theoretical prediction holds for most cases except the PVDF-NMP system, which was practically found to be soluble (1g PVDF was soluble in 10 ml NMP), as is also evident from existing literature[2]. Moreover, the PC-DMF system was practically poorly/sparingly soluble for concentrations as low as 1 g PC in 10 ml DMF.

Table S1

a) **Solubility results of the selected polymers in various solvents by visual inspection**

	**DMF**	**THF**	**$CHCl_3$**	**NMP**
**PVDF**	✔	✖	✖	✔
**PMMA**	✔	✔	✔	✔
**PC**	✖ (sparsely soluble)	✔ (soluble but as solute content increases, solubility drastically decreases)	✔	✔

b) **Hansen solubility parameter (HSP) and its parts for the studied polymers and solvents** [1]

Note that units used are $MPa^{1/2}$ for the HSP.

	**Dispersion (δ_d)**	**Polar (δ_p)**	**Hydrogen Bonding (δ_h)**	**Interaction Radius (R_0)**
**PVDF**	17.00	12.10	10.20	4.10

**PMMA**	18.64	10.52	7.51	8.59
**PC**	19.10	10.90	5.10	12.10
**DMF**	17.4	13.7	11.3	
**THF**	16.8	5.7	8.0	
**$CHCl_3$**	17.8	3.1	5.7	
**NMP**	17.0	2.8	6.9	

c) **Relative energy difference (RED) of various combinations and its interpretation based on the Hansen parameters***

	**RED**	**Conclusion**
**PVDF-DMF**	0.51	Soluble
**PVDF-THF**	1.65	Not Soluble
**PVDF-$CHCl_3$**	2.48	Not Soluble
**PVDF-NMP**	2.39	Not Soluble
**PMMA-DMF**	0.64	Soluble
**PMMA-THF**	0.71	Soluble
**PMMA-$CHCl_3$**	0.91	Soluble
**PMMA-NMP**	0.98	Soluble / partially soluble
**PC-DMF**	0.63	Soluble
**PC-THF**	0.62	Soluble
**PC-$CHCl_3$**	0.68	Soluble
**PC-NMP**	0.77	Soluble

* Note- Hansen solubility parameters may not hold for all systems and might have its limitation.

2) Characterization of polymers

This section includes the characterization of neat polymer for reference. Figure S2 shows the Fourier transform infrared (FTIR) spectra of commercial PVDF, PMMA and PC. The peaks corresponding to signature functional groups are highlighted in the respective polymers' FTIR spectra, based on the existing literature [3-9]. The observed spectra confirm that the materials under test are neat PVDF, PMMA and PC. The XRD of commercial PVDF used in this work can be obtained from the existing article published by Bose et al. [10]. The DSC thermogram of commercial PVDF and PC used in this work is already reported by Bose et al. [9, 10]. DSC was performed for PMMA and the glass transition temperature was observed to be ~100 °C.

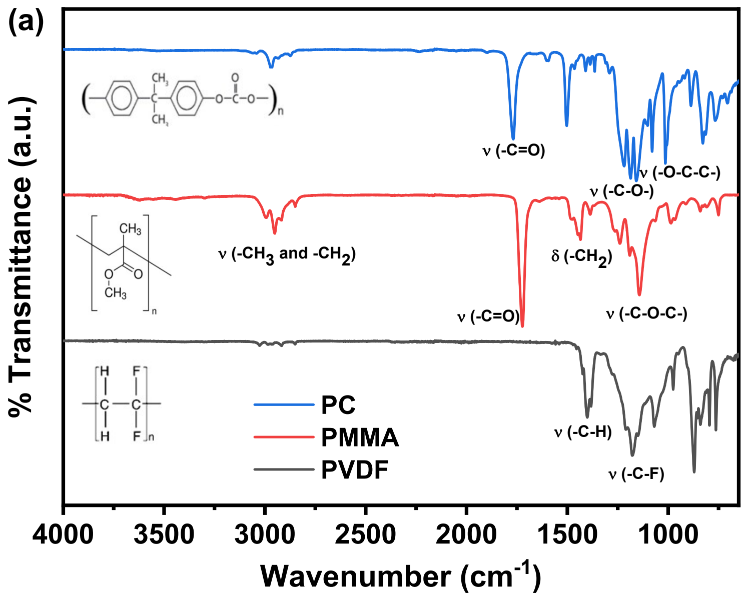

Figure S2: FTIR spectra of PC, PMMA and PVDF

3) Characterization of hybrid nanostructures

Figures S3a and S3b show the low and high magnification SEM micrographs of MoS_2-Fe_3O_4, whereas Figures S3c and S3d show those of rGO-Fe_3O_4.

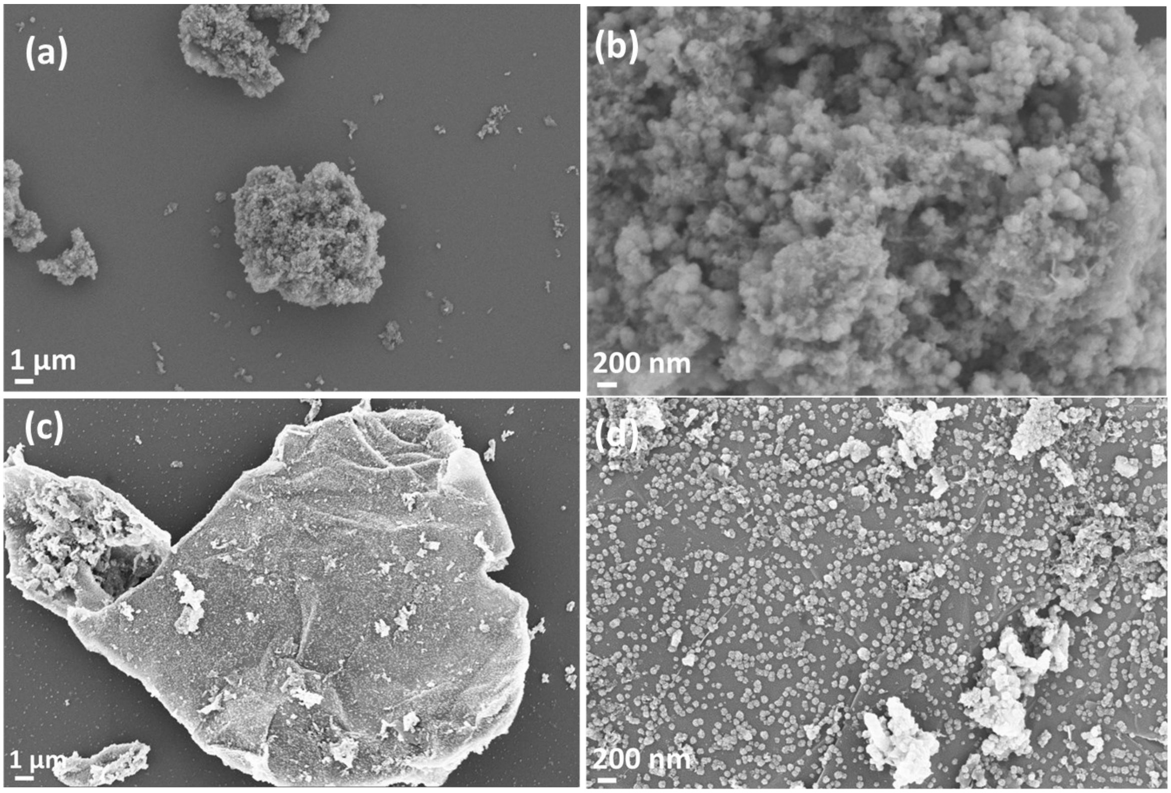

Figure S3: High and low magnification SEM micrographs of (a,b) MoS_2-Fe_3O_4 and (c,d) rGO-Fe_3O_4.

Figures S4a and S4b show the low magnification TEM micrograph of MoS_2-Fe_3O_4 and rGO-Fe_3O_4, whereas Figures S4c and S4d show the SAED pattern of both materials. The diffraction rings in the SAED pattern of rGO-Fe_3O_4 (figure S4d) conforms to the XRD pattern. However, the SAED pattern of MoS_2-Fe_3O_4 (figure S4c) comprises amorphous rings, which are probably because of lower crystallinity, which is in accordance with the XRD pattern where the peaks intensity is comparatively weaker than that of rGO-Fe_3O_4.

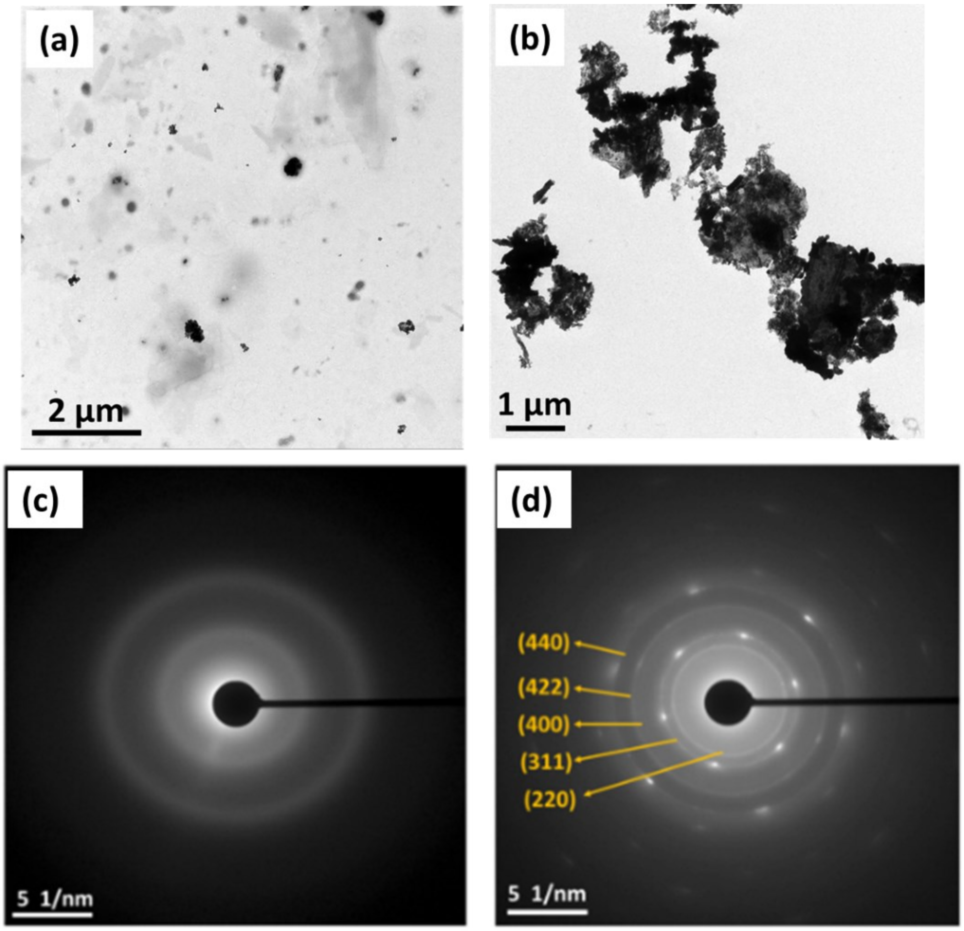

Figure S4: (a) Low magnification TEM micrographs of MoS_2-Fe_3O_4, (b) Low magnification TEM micrographs of rGO-Fe_3O_4, (c) SAED pattern of MoS_2-Fe_3O_4 and (d) SAED pattern of rGO-Fe_3O_4.

Figure S5 shows the digital images of PVDF (rGO-Fe_3O_4), PVDF (MoS_2-Fe_3O_4), PC(CNT), PVDF(rGO-Fe_3O_4)/M/PC, and PVDF(MoS_2-Fe_3O_4)/M/PC films obtained using the doctor blade method. It is to be noted that PVDF+ 10 wt% rGO-Fe_3O_4 obtained using doctor blade approach is designated as PVDF (rGO-Fe_3O_4), PVDF+ 10 wt% MoS_2-Fe_3O_4 is designated as PVDF (MoS_2-Fe_3O_4), PC+ 3 wt% CNT is designated as PC(CNT), the multilayered assembly with rGO-Fe_3O_4 is designated as PVDF(rGO-Fe_3O_4)/M/PC, and that with MoS_2-Fe_3O_4 as PVDF(MoS_2-Fe_3O_4)/M/PC. The thin films were obtained by casting and subsequent procedures, as shown in scheme 3. These digital images show that PVDF film, after incorporating 10 wt% nanofiller, resulted in a film with poor film quality. In contrast, the multilayered films obtained using this technique showed superior homogeneity and mechanical property. Though PC(CNT)

does not show a visible wear, it was structurally inferior for mechanical testing because of PC composite's brittle nature at such low thickness.

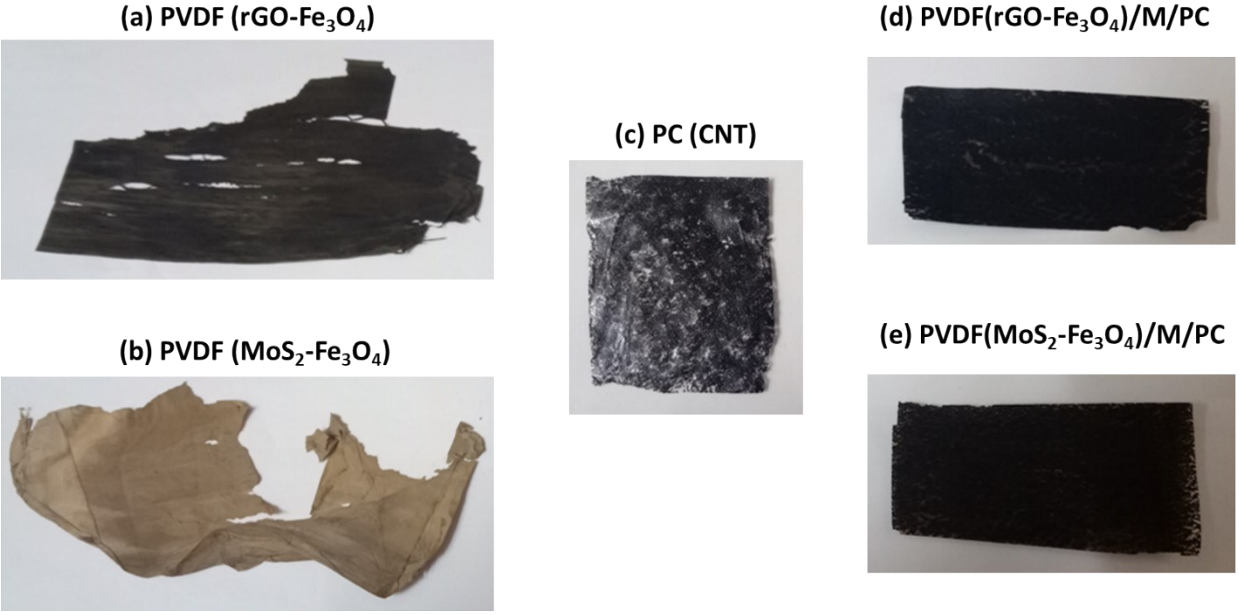

Figure S5: Digital images of (a) PVDF (rGO-Fe_3O_4) film, (b) PVDF (MoS_2-Fe_3O_4) film, (c) PC (CNT) film (d) PVDF(rGO-Fe_3O_4)/M/PC film, (e) PVDF(MoS_2-Fe_3O_4)/M/PC film obtained using the doctor blade method.

Figure S6 shows the EDS mapping of PVDF(MoS_2-Fe_3O_4)/M/PC. The sample was prepared with a rough approach. It was cut using a razor blade, and the cross-section was analyzed. The main idea was to understand the circular light grey regions in the SEM micrograph. From the EDS mapping shown in figure S6d, it is evident that those light grey regions are PVDF. It is also noted that these light grey circular regions are more prominent in samples cut by a razor blade than in samples prepared by ultramicrotome. Hence, we chose the approach using a razor blade for the analysis here. These light grey regions can be the crystallites of PVDF or are manifested during the fracture process towards the side of PVDF. Nevertheless, the area of this light grey matter is PVDF.

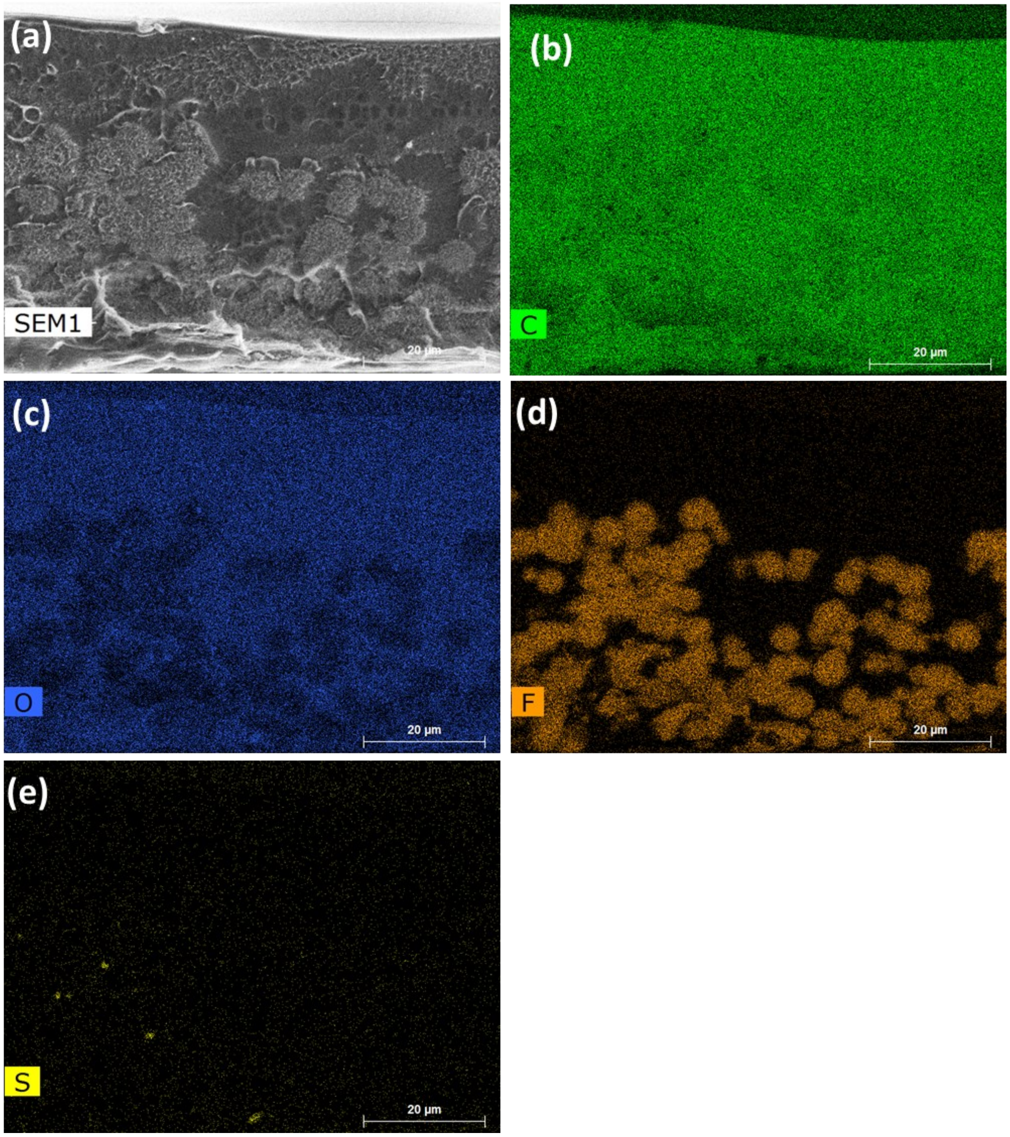

Figure S6: a) SEM micrograph of razor blade cut sample of PVDF(MoS_2-Fe_3O_4)/M/PC b) EDS mapping of carbon c) EDS mapping of oxygen d) EDS mapping of fluorine e) EDS mapping of Sulphur.

4) Additional dielectric and EM shielding results

Figure S7 shows the real permittivity of PC(CNT), PVDF(rGO-Fe_3O_4)/M/PC, and PVDF(MoS_2-Fe_3O_4)/M/PC films. The given samples real permittivity is in the frequency range of 10^{-1}- 10^7 is positive. By lowering the frequency, the real permittivity rises. The found result of PC(CNT) is quite different from the result reported before by our group [11], where the real permittivity showed negative values at low frequency. This indicates that the real permittivity is dependent not only

on the thickness of the sample but also on the concentration and state of dispersion of the nanoparticles in the PC matrix.

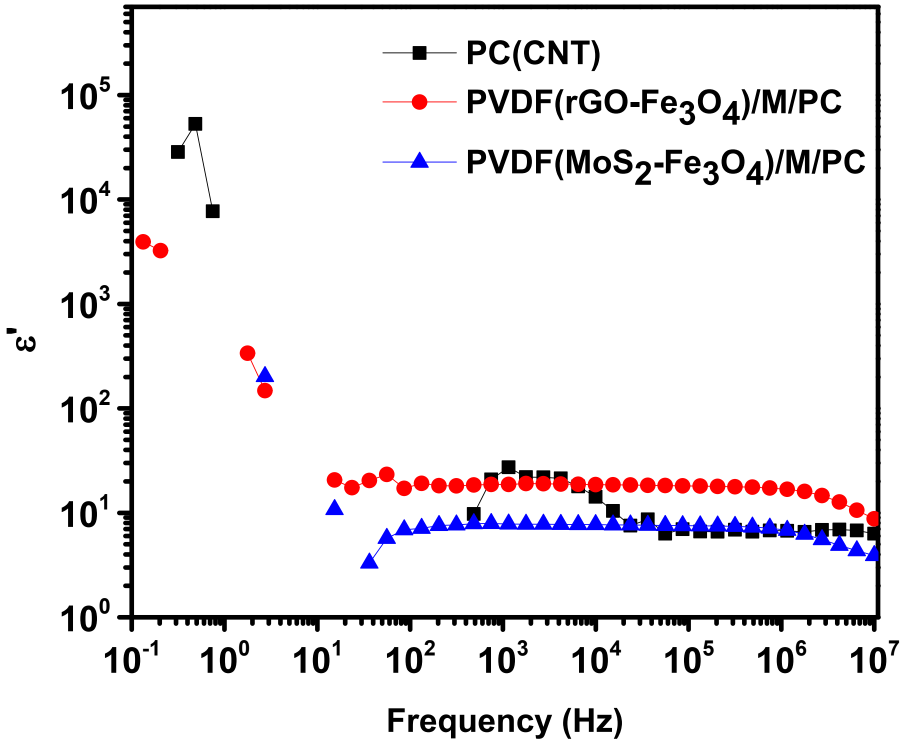

Figure S7: Real permittivity versus frequency.

Figures S8 shows SE_T as a function of frequency for one stack of PVDF+ 10 wt% rGO-Fe_3O_4 {or [PVDF(rGO-Fe_3O_4)]^1} and PVDF+ 10 wt% MoS_2-Fe_3O_4 {or [PVDF(MoS_2-Fe_3O_4)]^1}. It is to be noted that the superscript in the nomenclature denotes the number of stacks (here it is 1). SE_T is found to be -0.85 dB for [PVDF(rGO-Fe_3O_4)]^1 and -0.01 dB for [PVDF(MoS_2-Fe_3O_4)]^1 at 26.5 GHz.

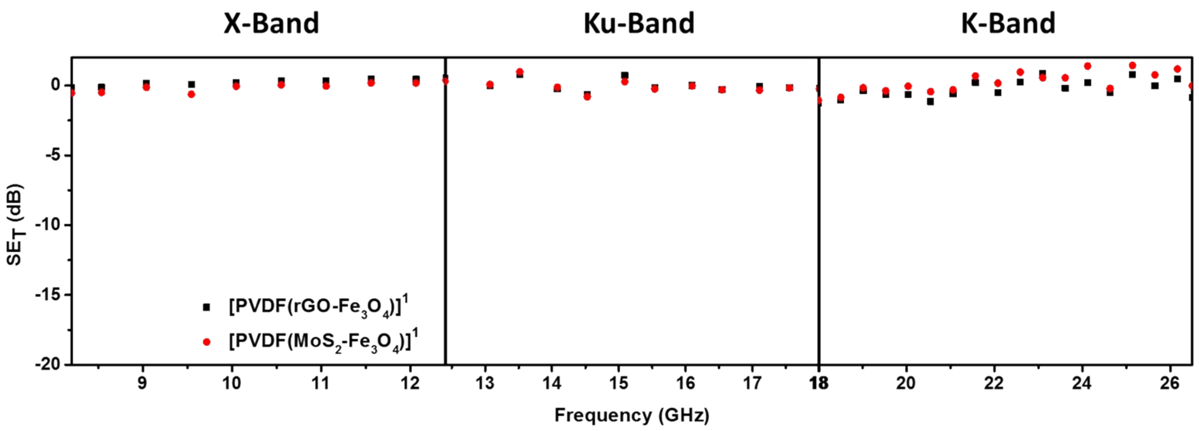

Figure S8: SE_T versus frequency for $[PVDF(rGO\text{-}Fe_3O_4)]^1$ and $[PVDF(MoS_2\text{-}Fe_3O_4)]^1$ in X band, Ku band, and K band.

Figures S9 illustrates the percentage of absorption/ reflection for $[PC(CNT)]^1$, $[PVDF(rGO\text{-}Fe_3O_4)/M/PC]^1$, and $[PVDF(MoS_2\text{-}Fe_3O_4)/M/PC]^1$ at 8.2 and 26.5 GHz frequency. The percentage of absorption for $[PC(CNT)]^1$ is found to be 71.9, $[PVDF(rGO\text{-}Fe_3O_4)/M/PC]^1$ is found to be 81.9, and $[PVDF(MoS_2\text{-}Fe_3O_4)/M/PC]^1$ is found to be 76.4 at 26.5 GHz frequency. The percentage absorption for $[PC(CNT)]^1$ is higher compared to $[PC(CNT)]^9$. As CNTs amount is 9-fold when using $[PC(CNT)]^9$, reflection-based shielding overpowered the absorption-based shielding [11]. However, this is not the case for the multilayered assemblies of $PVDF(rGO\text{-}Fe_3O_4)/M/PC$ and $PVDF(MoS_2\text{-}Fe_3O_4)/M/PC$, where absorption-based shielding dominates even on increasing the number of layers.

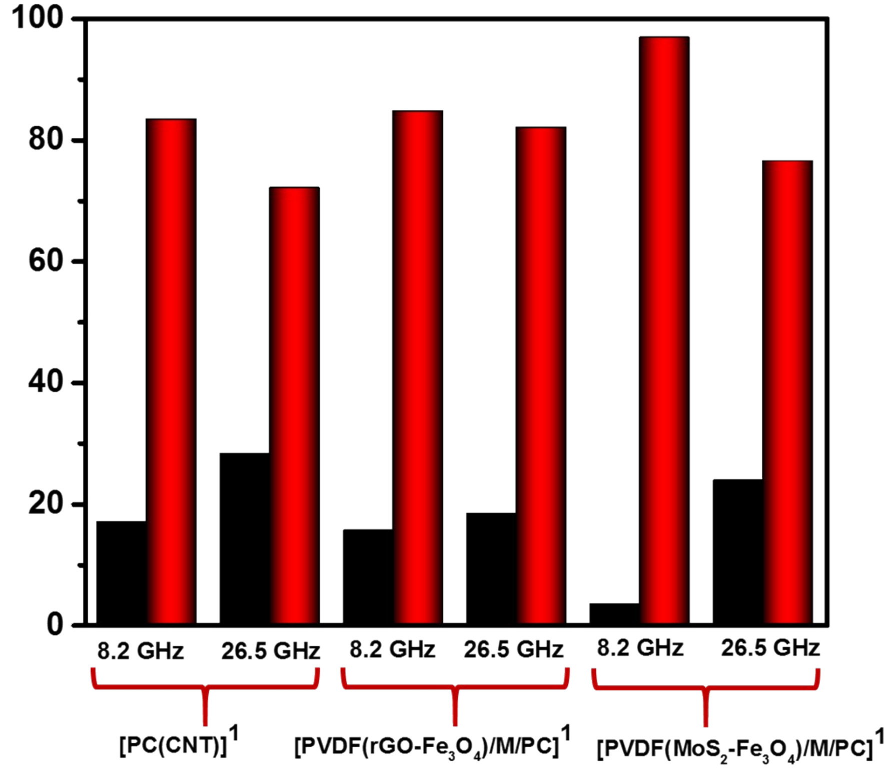

Figure S9: SE_A and SE_R for [PC(CNT)][1], [PVDF(rGO-Fe_3O_4)/M/PC][1] and [PVDF(MoS_2-Fe_3O_4)/M/PC][1] at 8.2 and 26.5 GHz frequency.

References

1. C. M. Hansen, *Hansen Solubility Parameters: A User's Handbook, Second Edition*, CRC Press, 2007.
2. J. Chang, J. Zuo, L. Zhang, G. S. O'Brien and T.-S. Chung, *J. Membr. Sci.*, 2017, **539**, 295-304.
3. Z. Zeng, D. Yu, Z. He, J. Liu, F.-X. Xiao, Y. Zhang, R. Wang, D. Bhattacharyya and T. T. Y. Tan, *Scientific Reports*, 2016, **6**, 20142.
4. D. Sugumaran and K. J. E. C. Karim, 2017, **2**, 1-11.
5. G. Duan, C. Zhang, A. Li, X. Yang, L. Lu and X. Wang, *Nanoscale research letters*, 2008, **3**, 118-122.
6. F. J. Tommasini, L. d. C. Ferreira, L. G. P. Tienne, V. d. O. Aguiar, M. H. P. d. Silva, L. F. d. M. Rocha and M. d. F. V. J. M. R. Marques, 2018, **21**.
7. B. S. Banerjee, S. S. Khaira and B. K, *RSC Advances*, 2014, **4**, 63380-63386.
8. B. C. Smith, The C=O Bond, Part VII: Aromatic Esters, Organic Carbonates, and More of the Rule of Three, https://www.spectroscopyonline.com/view/co-bond-part-vii-aromatic-esters-organic-carbonates-and-more-rule-three).
9. S. P. Pawar, 2018.
10. A. Gebrekrstos, G. Prasanna Kar, G. Madras, A. Misra and S. Bose, *Polymer*, 2019, **181**, 121764.
11. K. Sushmita, A. V. Menon, S. Sharma, A. C. Abhyankar, G. Madras and S. Bose, *The Journal of Physical Chemistry C*, 2019, **123**, 2579-2590.
